# Computational approaches identify a transcriptomic fingerprint of drug-induced structural cardiotoxicity

**DOI:** 10.1007/s10565-024-09880-7

**Published:** 2024-06-28

**Authors:** Victoria P.W. Au Yeung, Olga Obrezanova, Jiarui Zhou, Hongbin Yang, Tara J. Bowen, Delyan Ivanov, Izzy Saffadi, Alfie S. Carter, Vigneshwari Subramanian, Inken Dillmann, Andrew Hall, Adam Corrigan, Mark R. Viant, Amy Pointon

**Affiliations:** 1 Safety Sciences, Clinical Pharmacology & Safety Sciences, R&D, AstraZeneca, Cambridge, UK; 2 Phenomics, Data Sciences & Quantitative Biology, R&D AstraZeneca, Cambridge, UK; 3 Imaging and Data Analytics, Clinical Pharmacology & Safety Sciences, R&D, AstraZeneca, Cambridge, UK; 4 School of Biosciences, University of Birmingham, Edgbaston, Birmingham, UK; 5 Centre for Molecular Informatics, Department of Chemistry, University of Cambridge, Cambridge, UK; 6 High-Throughput Screening, R&D, AstraZeneca, Alderley Park, UK; 7 Imaging and Data Analytics, Clinical Pharmacology & Safety Sciences, R&D, AstraZeneca, Gothenburg, Sweden; 8 Disease Molecular Profiling, Discovery Biology, R&D AstraZeneca, Gothenburg, Sweden; 9 Phenome Centre Birmingham, University of Birmingham, Edgbaston, Birmingham, UK

**Keywords:** Structural cardiotoxicity, Calcium transients, Transcriptomics, Bioinformatics, Machine learning

## Abstract

Structural cardiotoxicity (SCT) presents a high-impact risk that is poorly tolerated in drug discovery unless significant benefit is anticipated. Therefore, we aimed to improve the mechanistic understanding of SCT. First, we combined machine learning methods with a modified calcium transient assay in human-induced pluripotent stem cell-derived cardiomyocytes to identify nine parameters that could predict SCT. Next, we applied transcriptomic profiling to human cardiac microtissues exposed to structural and non-structural cardiotoxins. Fifty-two genes expressed across the three main cell types in the heart (cardiomyocytes, endothelial cells, and fibroblasts) were prioritised in differential expression and network clustering analyses and could be linked to known mechanisms of SCT. This transcriptomic fingerprint may prove useful for generating strategies to mitigate SCT risk in early drug discovery.

**Graphical Abstract:**

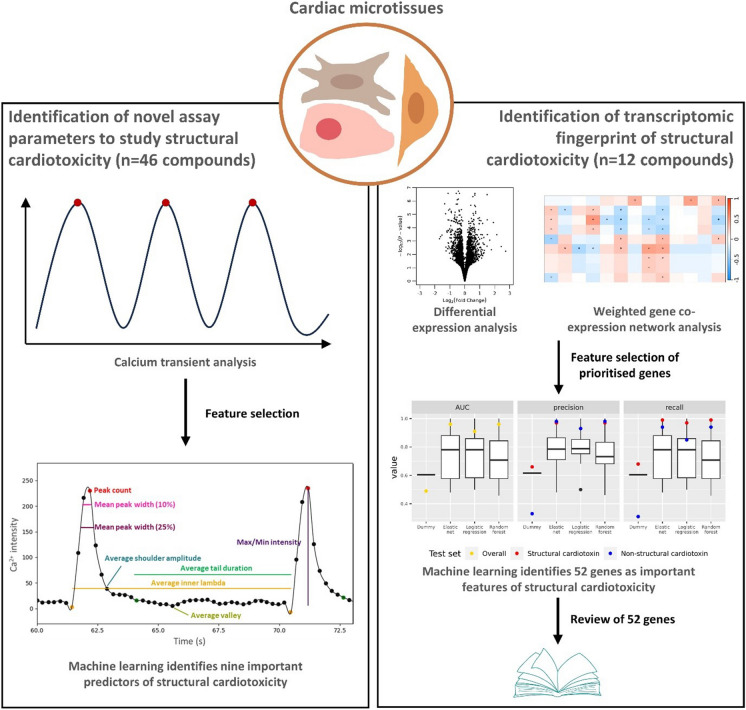

**Supplementary Information:**

The online version contains supplementary material available at 10.1007/s10565-024-09880-7.

## Introduction

Drug-induced structural cardiotoxicity (SCT) is clinically-defined as changes in left ventricular ejection fraction which may lead to fibrosis, cardiomyopathy, heart failure, and death. SCT presents a high-impact risk that limits clinical utility of novel therapies. Consequently, it is important to detect and mitigate SCT during the early stages of drug discovery to enable the development of therapies without this liability. SCT is defined as morphological damage of cardiac tissue and changes to intracellular organelles which clinically result in a decrease in left ventricular ejection fraction and heart failure (Laverty et al. 2011; Pointon et al. 2013).

Structural cardiotoxins including anti-cancer compounds such as anthracyclines (Swain et al. 2003; von Hoff et al. 1979), chemotherapeutic agents (Sara et al. 2018), and tyrosine kinase inhibitors (Crisci et al. 2019; Yamaoka et al. 2018) (Crisci et al. 2019; Sara et al. 2018; Swain et al. 2003; von Hoff et al. 1979; Yamaoka et al. 2018), have been associated with multiple phenotypic mechanisms, including oxidative stress (Geisberg and Sawyer 2010; Ott et al. 2007; S. Zhang et al. 2012), inflammation, apoptosis (Geisberg and Sawyer 2010; Ott et al. 2007; Youle and Van Der Bliek 2012), contractile dysfunction (Billingham et al. 1978; Geisberg and Sawyer 2010), extracellular matrix organisation and intercellular interactions (Hedin et al. 1997; Lorusso et al. 2020; Warn-Cramer and Lau 2004), and dysregulation of energy metabolism (Zhang et al. 2012) (Billingham et al. 1978; Geisberg and Sawyer 2010; Hedin et al. 1997; Lorusso et al. 2020; Ott et al. 2007; Warn-Cramer and Lau 2004; Youle and Van Der Bliek 2012; Zhang et al. 2012). These mechanisms have been linked to key organelles, for example mitochondria (Archer et al. 2018; Ott et al. 2007; Varga et al. 2015; Youle and Van Der Bliek 2012) and endoplasmic reticulum (ER) (Archer et al. 2018), but the molecular mechanisms and cellular drivers underpinning these changes remain unknown (Archer et al. 2018; Ott et al. 2007; Varga et al. 2015; Youle and Van Der Bliek 2012).

The development of humanised *in vitro* cardiac models provides an opportunity to study SCT by shedding light on the molecular mechanisms underlying phenotypic perturbations. Studies of human-induced pluripotent stem cell-derived cardiomyocytes (hiPS-CMs) and co-culture models (i.e. human cardiac microtissues) have shown that these cell models are sensitive to compound-induced changes in cardiomyocyte morphology and function (Karakikes et al. 2015; Pointon et al. 2013), and can be used to develop phenotypic assays for the detection of SCT (Archer et al. 2018) and molecular insights underlying these changes (Brandão et al. 2022; Chaudhari et al. 2016; Deidda et al. 2019; Glaab et al. 2021; Matsa et al. 2016; McSweeney et al. 2019; Palmer et al. 2020; Schmidt et al. 2023; van Hasselt et al. 2020; Yuan et al. 2020).

Phenotypic assays include assessment of key organelles including ER integrity and mitochondrial membrane potential (MMP) (Archer et al. 2018), previously reported as a predictive approach for SCT and physiological processes. For example, the calcium transient assay,^2^ when combined with the software CardioWave (Yang et al. 2022), can derive up to 40 parameters. These parameters have been used to build machine learning models to study acute cardiotoxicity (Yang et al. 2022).

Molecular insights can be gained via ‘omics’ profiling to identify mechanistic markers of toxicity. To date, transcriptomic (Chaudhari et al. 2016; Glaab et al. 2021; Matsa et al. 2016; McSweeney et al. 2019; van Hasselt et al. 2020), metabolomic (Deidda et al. 2019; Palmer et al. 2020; Yuan et al. 2020), and proteomic (Brandão et al. 2022; Schmidt et al. 2023; Yuan et al. 2020) technologies, applied separately and *in tandem,* have identified several hallmark pathways of general cardiotoxicity. As highlighted in observational and clinical studies, pathways identified in these studies include, but are not limited to, cell adhesion, energy metabolism, oxidative stress, calcium homeostasis and contractility, protein homeostasis, apoptosis, and mitochondrial homeostasis (Brandão et al. 2022; Chaudhari et al. 2016; Deidda et al. 2019; Glaab et al. 2021; Matsa et al. 2016; McSweeney et al. 2019; Palmer et al. 2020; Schmidt et al. 2023; van Hasselt et al. 2020; Yuan et al. 2020). Despite the broad range of pathways and molecular players identified, these studies primarily focus on assessing a limited range of anthracyclines or tyrosine kinase inhibitors (TKIs) (Brandão et al. 2022; Chaudhari et al. 2016; Deidda et al. 2019; Glaab et al. 2021; Matsa et al. 2016; McSweeney et al. 2019; Palmer et al. 2020; Schmidt et al. 2023; van Hasselt et al. 2020; Yuan et al. 2020).

Furthermore, studies performed *in vitro* have focused on single cell types, predominantly cardiomyocytes, while ignoring other cellular constitutes in the heart that represents 70% of the myocardium cell mass. Functional evidence exists to support the role of non-cardiomyocytes in SCT. For example, cardiac fibroblasts may regulate cardiomyocyte function by secreting paracrine signaling factors such as TGF-beta and interleukin-6, which in turn can lead to cardiac hypertrophy and electrophysiological changes (Cartledge et al., 2015). In SCT, these processes may be disrupted, leading to cardiac fibrosis. Another example is the cross-talk between endothelial cells and cardiomyocytes. Endothelial cells may secrete endothelin-1, which binds to cardiomyocyte endothelin-1 receptors to promote cardiomyocyte survival (Schorlemmer et al., 2008). With SCT, endothelial cell dysfunction may thus lead to cardiomyocyte apoptosis and cardiac degeneration. Other mechanisms are reviewed more comprehensively elsewhere (Guo et al., 2021), nevertheless, the study of additional cardiac cell types in a human-based *in vitro* model system may reveal physiologically-relevant and novel pathways of SCT.

**Fig. 1:**
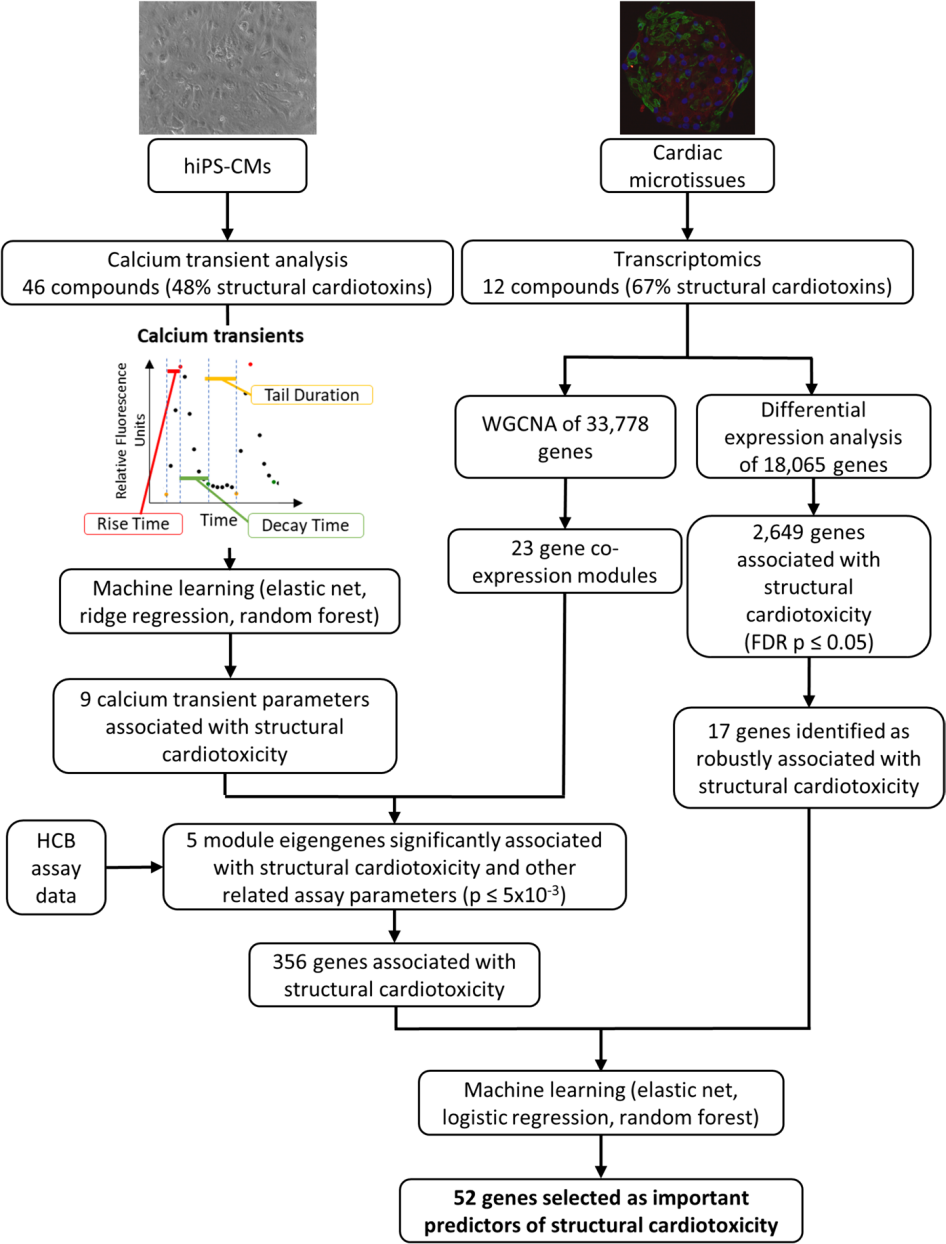
Study design. (Top left) Calcium transient assays were performed on hiPS-CMs derive parameters across 46 compounds (48% structural cardiotoxins). Machine learning was then used to select 9 calcium transient parameters as important putative predictors of SCT. (Top right) Transcriptomic analysis was performed on cardiac microtissues exposed to an overlapping set of 12 compounds (67% structural cardiotoxins). Differential expression and network clustering approaches were used to prioritise genes as features for machine learning classifiers of SCT, and feature selection was performed to identify a list of 52 gene predictors for SCT. HCB = High Content Biology

Here, we use phenotypic assay data in cardiomyocytes and transcriptomic data in cardiac microtissues to i) identify phenotypic parameters associated with SCT; ii) characterise the gene expression changes in response to several known structural cardiotoxins, and iii) identify a molecular fingerprint of SCT (Fig. 1).

## Results

### Identification and characterisation of calcium transient parameters associated with SCT

Calcium transient parameters were calculated from the calcium transients of hiPS-CMs exposed to 46 compounds (Supplementary Table 1; Supplementary Experimental Procedures). Of 40 parameters derived from CardioWave, 16 described the standard errors of other parameters and were not included due to limited interpretability compared to their corresponding mean parameter measures. The remaining 24 were unique parameters which described measures of peak count, amplitude, and other features of the calcium transient waveform including peak width, shoulder, tail, and valley (Supplementary Table 1).

Of the 24 parameters considered, we included 17 which were uncorrelated (Pearson R^2^ < 0.80) as features to assess seven machine learning classifiers of SCT. Compared to a dummy classifier, all models performed better at predicting SCT by area under curve (AUC) (Supplementary Figure 1). The performance across machine learning models on predicting SCT was moderate across models (from AUC = 0.64 in Gaussian Naïve Bayes to AUC = 0.85 in logistic regression), in line with the expectation that calcium transients alone do not fully capture mechanisms of SCT.

**Fig. 2:**
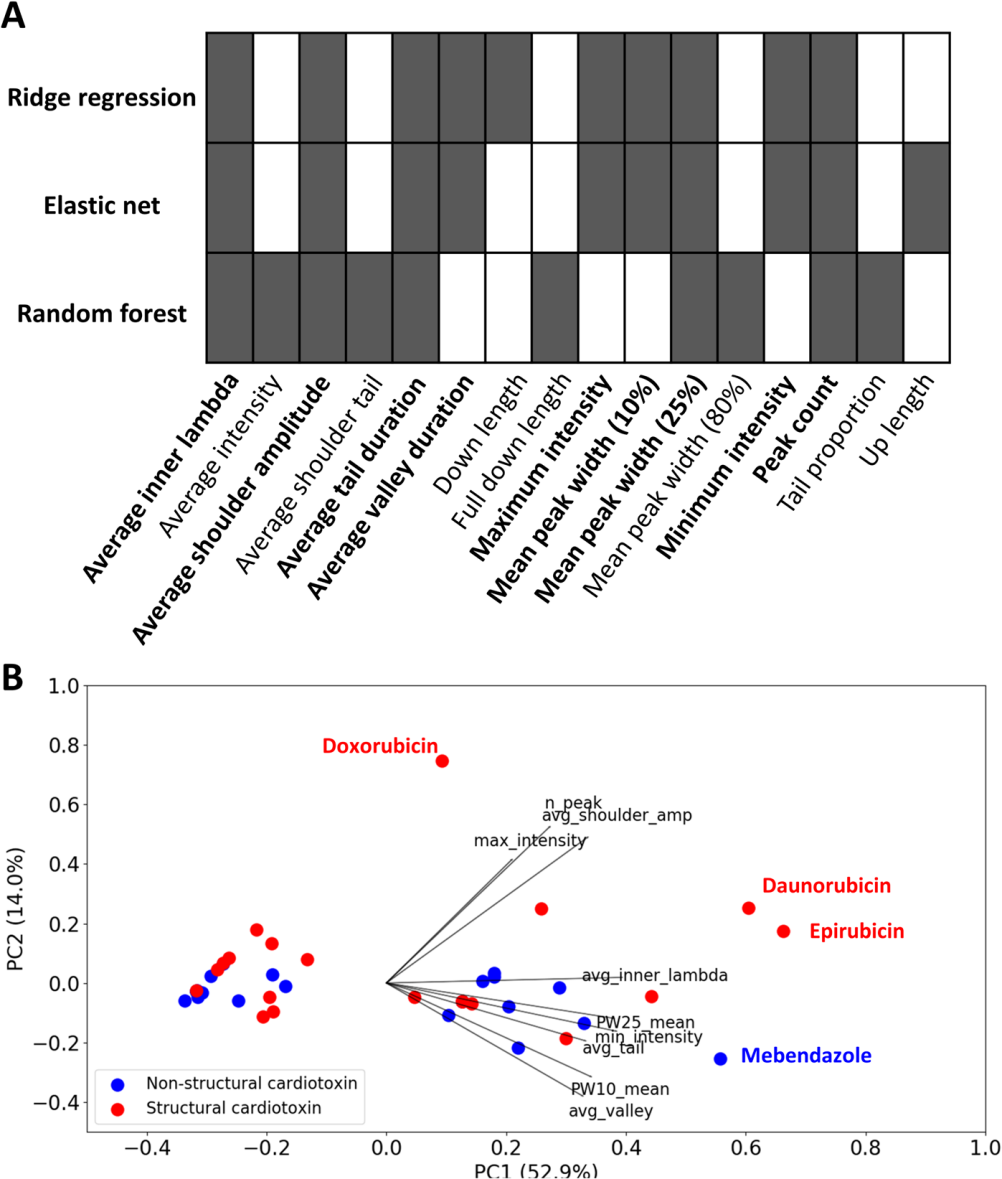
Identification of waveform parameters associated with SCT. **A**) Filled-in boxes and bolded parameter labels highlight parameters which were selected in two or more machine learning models of SCT. **B)** Principal component analysis of structural cardiotoxicity by the nine selected parameters. Some of the structural cardiotoxins emerged as distinct along PC2. avg_inner_lambda = average inner lambda; avg_shoulder_amp = average shoulder amplitude; avg_tail = average tail duration; avg_valley = average valley duration; max_intensity = maximum intensity; PW10_mean = mean peak width (10%); PW25_mean = mean peak width (25%); min_intensity = minimum intensity; n_peak = peak count

To identify important features associated with SCT, we examined the top ten important features highlighted in two selected linear models (ridge regression and elastic net) and one non-linear model (random forest). Nine features (average inner lambda, average shoulder amplitude, average tail duration, average valley duration, maximum intensity, minimum intensity, mean peak width (10%), mean peak width (25%), peak count) were highlighted as important features of SCT in at least two of these models (Fig. 2). These features accounted for some compound-specific variation in the dataset (Fig. 2B). A logistic regression classifier of the 9 features performed better at distinguishing structural cardiotoxins from non-structural cardiotoxins than a baseline classifier including only peak count and average amplitude as features (Table 1). These results indicate the importance of leveraging multiple phenotypic assays and parameters to capture non-overlapping mechanisms of SCT.

**Table 1:** A logistic regression classifier of nine selected calcium transient features performs better at distinguishing structural cardiotoxins from non-structural cardiotoxins than a baseline classifier. Ten times repeated, five-fold internal cross-validation was performed on the 46 clinical compounds

Logistic regression classifier	Dataset	Area Under Curve (min-max)	Precision (min-max)	Recall (min-max)
Baseline	Cross-validation	0.61 (0.30 – 1)	0.60 (0.21 – 1)	0.61 (0.30 – 1)
Baseline	Test	0.65	0.69	0.50
9-parameter	Cross-validation	0.71 (0.30 – 1)	0.75 (0.21 – 1)	0.71 (0.30 – 1)
9-parameter	Test	0.80	0.88	0.68

### Characterisation of the effects of structural and non-structural cardiotoxins on the gene expression profiles of cardiac microtissues

Transcriptomic profiling was performed in cardiac microtissues using 12 compounds (67% structural cardiotoxins) at three timepoints (6 hr, 48 hr, 72 hr) and two concentrations (Supplementary Table 2; Experimental Procedures). High concentrations were determined for each compound based on the lowest concentration of either i) IC_30_ of ATP depletion in cardiac microtissues in a high content biology assay associated with SCT; ii) ≤15x total C_max_ (to maintain therapeutic relevance); or iii) lowest investigated concentration (Archer et al. 2018). Low concentrations were calculated as the half-log dilution of the high concentrations.

A total of 51,195 genes were measured across samples, of which 18,065 with sufficient gene counts and variance were assessed for differential gene expression analysis (Experimental Procedures). After adjusting for batch effects, we found that gene expression profiles were driven by compound-specific effects as well as smaller effects induced by timepoint and concentration (Supplementary Figure 2).

Across the 12 compounds assessed, the number of differentially expressed genes (absolute log2(Fold-Change) > 1, FDR *p* ≤ 0.05) ranged from 0 to 2,838 (Supplementary Table 3). Compounds with similar gene expression profiles may indicate similar molecular mechanisms of cardiotoxicity; however, compounds exhibit different potencies at different concentrations. To account for these differences, we selected the treatment with the largest number of differentially expressed genes for each compound for analysis. Hierarchical clustering based on the 4,292 genes which were significantly differentially expressed following exposure to one or more compounds identified four clusters (Supplementary Figure 3). Of these, two clusters had sufficient numbers of differentially expressed genes to provide evidence of pathway enrichment. One cluster, comprising erlotinib (48 hr high), idarubicin (48 hr high), and fluorouracil (48 hr high) (Supplementary Figure 3), had 41 over- or under-represented pathways. Enriched pathways included tRNA charging and Rho GTPase inhibition pathways, while depleted pathways included immune response and signalling pathways (Supplementary Table 4). Another cluster comprised of sorafenib (48 hr high), dasatinib (48 hr high), and sunitinib (48 hr high) (Supplementary Figure 3). Over-representation analysis of differentially expressed genes across these treatments revealed 70 over- or under-represented pathways (FDR *p*-value ≤ 0.05, absolute(Z) ≥ 2). Pathways relating to DNA damage repair, cell cyclin regulation, and bile acid signalling and metabolism were up-regulated while those relating to apoptosis, IL-6/IL-8 mediated inflammation, and cell-cell adhesion signalling were down-regulated (Supplementary Table 4). Taken together, these findings suggested that compounds exerted much larger effects on gene expression profiles irrespective of SCT class, and we therefore explored alternative approaches to identify mechanisms of SCT.

### Differential expression analysis highlights 20 genes which are robustly associated with SCT

**Fig. 3:**
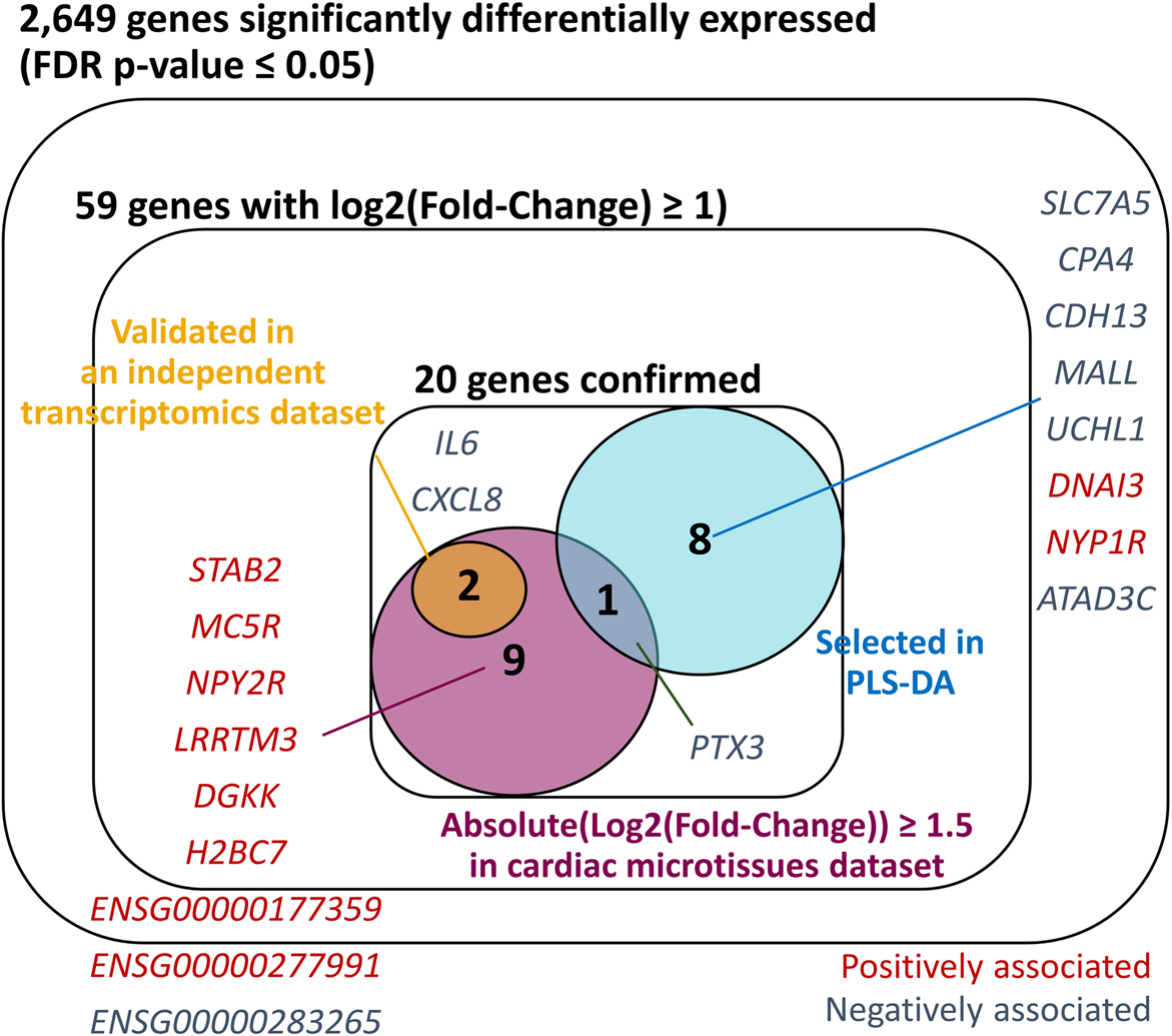
Prioritised genes from differential gene expression analysis. Prioritisation was performed using additional methods: i) validation in an independent transcriptomic dataset (van Hasselt et al. 2020); ii) partial least squares determinant analysis, and iii) introducing a stricter log_2_(Fold-Change) threshold (≥1.5) on differential expression analysis results. See main text and experimental procedures for more detail

Of 18,065 assessed genes, 2,649 were significantly differentially expressed in structural cardiotoxins compared to non-structural cardiotoxins (FDR *p* ≤ 0.05) (Fig. 3; Supplementary Table 5). Of the 2,649 genes, 1342 were down-regulated and 1307 were up-regulated. Over-representation analysis showed that the 2,649 genes were enriched in 222 pathways (Supplementary Table 6). The top 50 pathways related to cell-cell junctions and adhesion, extracellular matrix binding and organisation, endoplasmic reticulum stress and processes of protein folding and transport, and actin cytoskeleton remodelling (Supplementary Table 6).

Of the 2,649 genes which were differentially expressed in structural cardiotoxins compared to non-structural cardiotoxins, 59 also had an absolute effect size > 1 (Fig. 3; Supplementary Table 5). We performed further assessment of these 59 genes using the following:

1. Replication in an independent transcriptomic profiling dataset: Differential expression analysis of 5 structural cardiotoxins compared to 16 non-structural cardiotoxins using gene expression data from van Hasselt et al. (2020). A total of 224 genes were significantly differentially expressed (FDR-adjusted *p* ≤ 0.05), and 28 of these had an absolute effect size > 1. Of the 28 genes, 2 (*IL6* and *CXCL8*) were also among the 59 prioritised genes in the cardiac microtissue dataset.
2. Partial least squares determinant analysis (PLS-DA): Hyperparameter fine-tuning showed that 2 principal components were sufficient for PLS-DA, and good separation of structural cardiotoxins and non-structural cardiotoxins was observed (Supplementary Figure 4A; B). When ranked by absolute weighting, the weights of significant genes identified in differential expression analysis showed a skewed distribution towards higher ranks compared to non-significant genes (Supplementary Figure 4C). Of the top 100 genes, 9 were also highlighted in differential expression analysis (Fig. 3).
3. Genes were also prioritised if they had absolute log2(Fold-Change) values ≥ 1.5 in the differential expression analysis performed in cardiac microtissues. Of the 59 prioritised genes, 12 had absolute effect sizes which met this threshold.

Altogether, a total of 20 genes were robustly associated with SCT, and the 17 protein-coding genes among this set were prioritised for downstream analysis (Fig. 3). Single cell expression data from GTEx v8.0 (Aguet et al. 2020) suggests that many of these genes are expressed predominantly in endothelial cells or fibroblasts, highlighting the importance of studying non-cardiomyocytes to understand mechanisms of SCT. A qRT-PCR analysis of six of the significant genes (*ATAD3C*, *CDH13*, *IL6*, *PTX3*, *SLC7A5*, *UCHL1*), as detailed in the Supplementary Experimental Procedures, showed that treated cardiac microtissues showed mainly consistent down-regulation of these genes compared to control at high concentrations after 48 hour exposures, which corroborated findings from next generation sequencing (Supplementary Table 7; Supplementary Experimental Procedures).

### Complementary WGCNA analysis identifies hub genes in biologically-relevant modules associated with SCT

Co-expressed genes are more likely to represent common biological pathways than genes which are not co-expressed. To test whether measured genes formed biologically-relevant clusters representing SCT, we performed WGCNA, a co-expression network clustering approach (Langfelder and Horvath 2008), and tested the association of module eigengenes (MEs) with SCT and related parameters from the calcium transient and HCB assays (Supplementary Experimental Procedures). At 6 hrs, we observed few differentially expressed genes across compounds (Supplementary Figure 2). Therefore, we excluded samples at 6 hrs from WGCNA analysis.

A total of 23 co-expressed gene modules were identified across 33,778 genes (Supplementary Table 8). We used random colours to name the modules. The median number of genes in a module was 433 (salmon module); the paleturquoise module had the least number of genes at 35, while the turquoise module had the maximum number of genes at 12,250. The eigengenes of these modules correlated strongly with those from modules of WGCNA networks which included vehicle controls (Supplementary Figure 5).

**Fig. 4:**
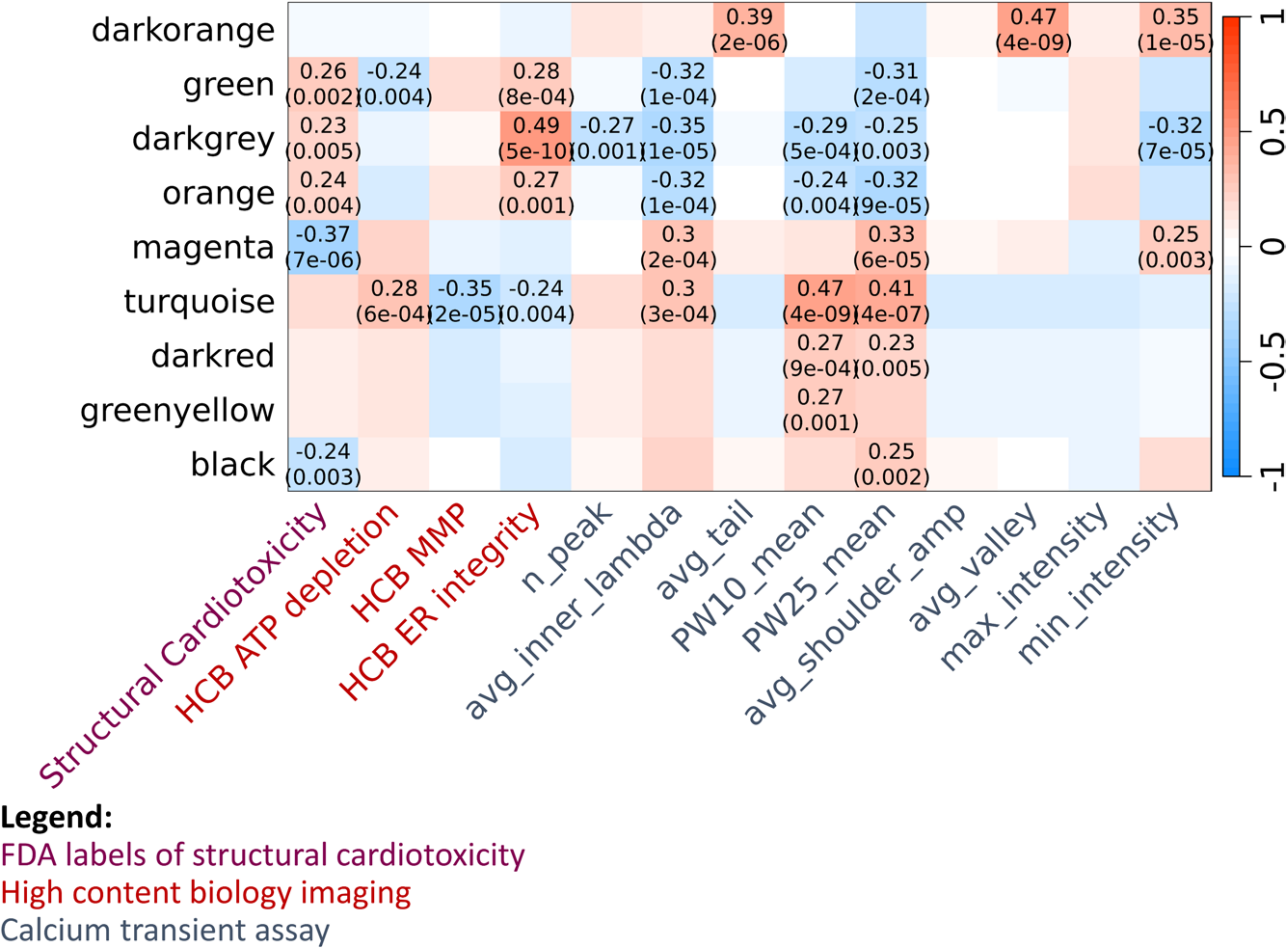
ME-trait associations with SCT and related phenotypic assay parameters. Of the 23 modules identified, 9 had at least one association with SCT or a phenotypic assay parameter. Values reported in the cells are Pearson’s R^2^ correlations with unadjusted p-values reported in brackets; only cells reaching significance (*p* ≤ 5x10^-3^) are labelled. N_peak = peak count; avg_inner_lambda = average inner lambda; avg_tail = average tail duration; PW10_mean = mean peak width (10%); PW25_mean = mean peak width (25%); avg_shoulder_amp = average shoulder amplitude; avg_valley = average valley duration; max_intensity = maximum intensity; min_intensity = minimum intensity

Of the 23 modules, 9 were associated with one or more tested phenotypes, and 5 of these (magenta, green, darkgrey, orange, and black) were associated with SCT at an adjusted p-value threshold of 5x10^-3^ (Fig. 4). The five modules associated with SCT showed strong and significant (*p* ≤ 0.05) correlations of gene significance for SCT with module membership (Supplementary Figure 6).

The green (*n*=2,511), darkgrey (*n*=207), and orange (*n*=731) modules were positively-associated with SCT and with HCB phenotypes, and inversely-correlated with several calcium transient parameters (Fig. 4). Over-representation analysis of genes within each of these modules, as detailed in the Supplementary Experimental Procedures, showed that these modules represented related functional pathways (Supplementary Table 9). The green and orange modules were over-representative of genes associated with mitochondrial inner membrane and mitochondria-mediated energy metabolism, while the darkgrey module was over-representative of pathways relating to muscle contraction and sarcoplasmic reticulum (Supplementary Table 9). Among these modules, only the green module contained two differential expression analysis-prioritised genes (*MC5R*, *DGKK*) (Supplementary Figure 6; Supplementary Table 8).

By contrast, the magenta (*n*=3,757) and black (*n*=1,120) modules were inversely-correlated with SCT and with HCB phenotypes, and positively-correlated with several calcium transient phenotypes (Fig. 4). Over-representation analysis showed that these modules were over-representative of pathways relating to extracellular matrix organisation and structure, focal adhesion, ubiquitin-mediated protein degradation, and integrity and activity of other structural cellular components (Supplementary Table 9). The magenta module also contained nine genes prioritised in differential expression analysis (*ATAD3C, CDH13, CPA4, CXCL8, IL6, MALL, UCHL1, PTX3, SLC7A5*) (Supplementary Table 8).

**Fig. 5:**
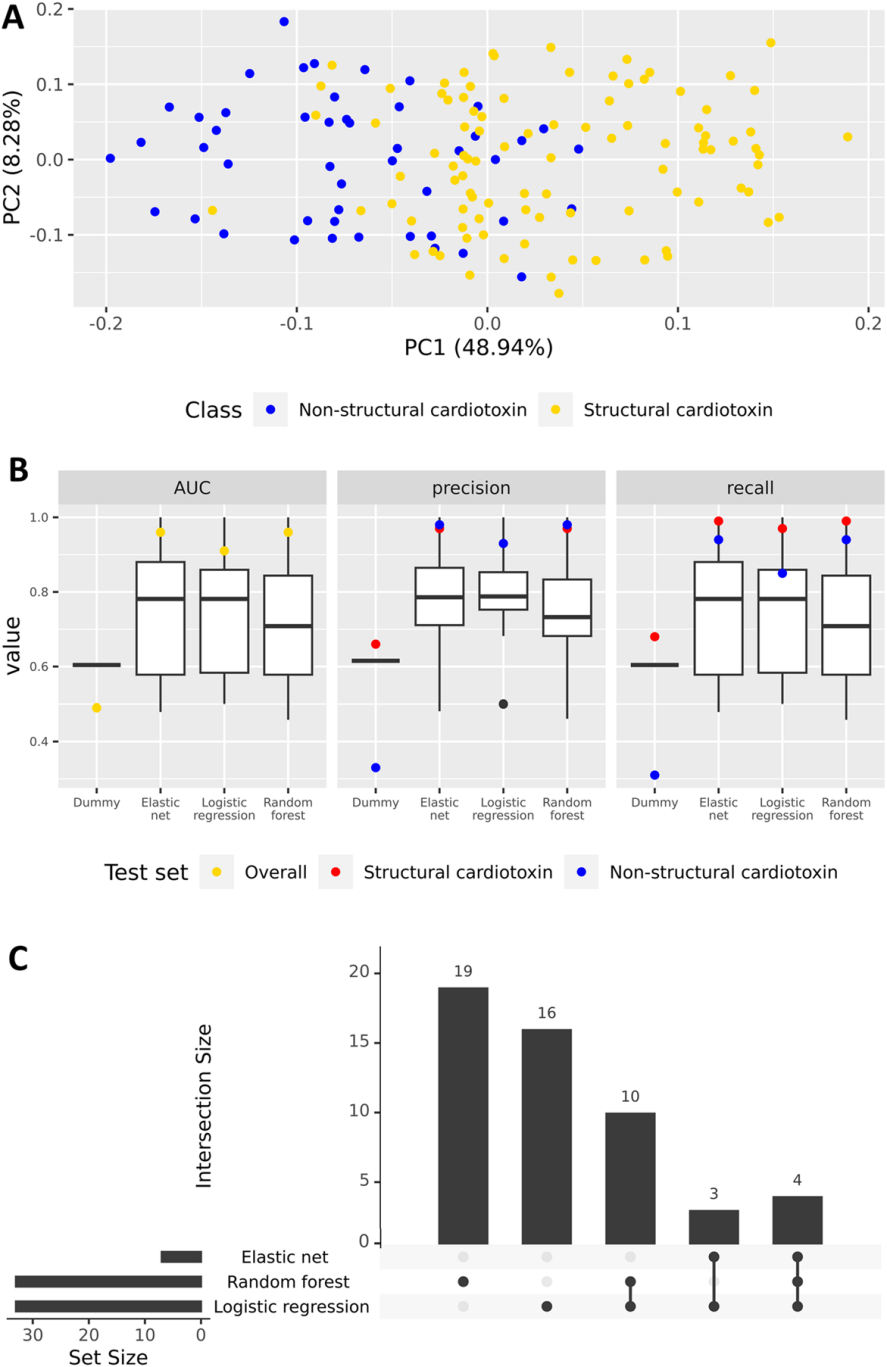
SCT classification using 367 prioritised genes. **A)** Principal component analysis of 367 prioritised genes. **B)** Area under curve (AUC), precision, and recall of elastic net, logistic regression, and random forest models. Box-and-whisker plots represent cross-validation performance. Yellow points represent AUC performance in the full test dataset while red and blue points represent performance of structural cardiotoxins and non-structural cardiotoxins, respectively. **C)** Intersection plot summarising the number of important features which are shared and unique to each machine learning model

Across the five modules implicated in SCT, we prioritised 353 genes with significant module membership (Bonferroni-corrected *p* ≤ 1.48x10^-6^, adjusting for 33,778 genes) (Supplementary Figure 6), and a further 3 by intra-modular connectivity (Supplementary Figure 7). After including genes prioritised by DESeq2, we obtained a list of 367 unique genes for downstream analysis (Supplementary Table 8). Principal component analysis showed that these genes could partially separate structural cardiotoxins from non-structural cardiotoxins along PC1 (Fig. 5A).

### Machine learning analysis prioritises 52 genes as important predictors of SCT

The 367 genes were uncorrelated (Pearson R^2^ < 0.95) and had a median variance of 0.068 (minimum = 0.0066, maximum = 1.61). Of the 367 genes, 328 had high variance (ơ^2^ ≥ 0.02) and were used as features in machine learning analysis. All three machine learning classifiers performed better (AUC ≥ 0.91, precision_structural cardiotoxin_ ≥ 0.93, recall_structural cardiotoxin_ ≥ 0.97) than a dummy classifier of SCT (AUC = 0.49, precision_structural cardiotoxin_ ≥ 0.66, recall_structural cardiotoxin_ ≥ 0.68) (Fig. 5B). Overall, the models had improved precision and recall for identifying structural cardiotoxins than non-structural cardiotoxins, which could be due to the class imbalance in the dataset (Fig. 5B).

Of the 328 gene predictors, 52 across SCT-associated modules were selected by one or more machine learning models as important features (Fig. 5C; Table 2; Supplementary Table 10), of which five (*DNAI3, LRRTM3, NPY1R, NPY2R, SLC7A5*) were also prioritised by differential expression analysis. Single cell expression data from the Human Protein Atlas (Karlsson et al. 2021) showed that 32 genes were more highly expressed or almost exclusively expressed in non-cardiomyocytes compared to cardiomyocytes (Supplementary Table 10). Based on this evidence and on existing literature, genes were putatively assigned to one or more of 13 pathways: ‘Angiogenesis’, ‘Apoptosis’, ‘Cardiac development’, ‘Contractility’, ‘Drug metabolism’, ‘ECG’, ‘ECM/Cell adhesion’, ‘Energy metabolism’, ‘ER processes’ (including protein synthesis and folding), ‘Fibrosis’, ‘Inflammation’, ‘Oxidative stress’, and ‘Transcription’ (Table 2; Supplementary Table 10). Seven of the genes could not be assigned to a pathway due to lack of evidence and were labelled as ‘Unknown’ (Table 2; Supplementary Table 10).

**Table 2:** Summary of 52 prioritised genes and pathways relating to SCT

HGNC symbol	WGCNA module	Putatively assigned pathways	Primary cell expressed, as per Human Protein Atlas (Supplementary Table 10)
*ALDH1L2*	black	Apoptosis, Oxidative stress	Cardiac fibroblasts
*ASXL3*	green	Cardiac development, Energy metabolism	Cardiomyocytes
*B4GALT4*	black	Unknown	Cardiomyocytes
*BEND6*	black	Transcription	Cardiomyocytes
*CARS1*	magenta	Oxidative stress	Endothelial cells
*COL12A1*	magenta	ECM/Cell adhesion, Fibrosis	Cardiac fibroblasts
*COL15A1*	magenta	ECM/Cell adhesion, Fibrosis	Cardiac fibroblasts
*DEPP1*	magenta	Apoptosis, Oxidative stress	Cardiomyocytes
*DNAI3*	brown	ECM/Cell adhesion	Cardiomyocytes
*EPHX1*	magenta	Energy metabolism, Inflammation	Cardiac fibroblasts
*FAM13C*	green	Energy metabolism	Endothelial cells
*FNDC3B*	black	Angiogenesis, Fibrosis	Cardiac fibroblasts
*FNDC4*	magenta	Cardiac development, Inflammation	Cardiac fibroblasts
*FOS*	magenta	ER processes, Oxidative stress, Apoptosis	Cardiomyocytes
*GCLM*	black	Apoptosis, Oxidative stress	Endothelial cells
*GRB10*	magenta	ECM/Cell adhesion, Cardiac development, Angiogenesis	Endothelial cells
*HDAC4*	magenta	Cardiac development, Transcription, Apoptosis	Cardiomyocytes
*HSD11B1*	magenta	Angiogenesis, ER processes, Cardiac development	Cardiac fibroblasts
*INKA2*	orange	ER processes	Cardiomyocytes
*ITGA8*	magenta	ECM/Cell adhesion	Endothelial cells
*JDP2*	magenta	Transcription	Cardiac fibroblasts
*KIAA1217*	darkgrey	Cardiac development	Endothelial cells
*LINC00536*	magenta	Unknown	Not found in database
*LPAR5*	magenta	Transcription	Cardiomyocytes
*LPCAT1*	magenta	Energy metabolism	Endothelial cells
*LRRTM3*	turquoise	Unknown	Cardiomyocytes
*LUCAT1*	black	Oxidative stress, Apoptosis, Inflammation	Not found in database
*MGST1*	magenta	Oxidative stress	Cardiac fibroblasts
*NPY1R*	turquoise	Contractility, Cardiac development	Endothelial cells/Cardiac fibroblasts
*NPY2R*	turquoise	Contractility, Cardiac development	Not detected in experiments
*NQO1*	magenta	Oxidative stress	Endothelial cells
*NTN1*	magenta	ECM/Cell adhesion, Oxidative stress	Cardiomyocytes
*OPN3*	black	Unknown	Endothelial cells
*OSMR*	magenta	ECM/Cell adhesion, Angiogenesis, Inflammation	Endothelial cells
*PGD*	magenta	Energy metabolism	Cardiac fibroblasts
*PLSCR4*	black	Energy metabolism	Endothelial cells
*PRSS35*	green	ECG	Cardiomyocytes
*RNF24*	magenta	Contractility	Cardiac fibroblasts
*RPS6KA3*	black	Contractility	Endothelial cells
*SARS1*	magenta	Angiogenesis, Cardiac development	Cardiomyocytes
*SEL1L3*	magenta	Unknown	Endothelial cells
*SLC1A5*	magenta	Energy metabolism	Cardiac fibroblasts
*SLC7A11*	black	Oxidative stress	Cardiomyocytes
*SLC7A5*	magenta	Angiogenesis	Cardiac fibroblasts
*SRXN1*	magenta	Oxidative stress	Endothelial cells
*STAG3L3*	orange	Unknown	Not found in database
*SULT1C4*	green	Drug Metabolism	Endothelial cells
*THSD4*	magenta	Apoptosis, Oxidative stress, ECM/Cell adhesion	Cardiomyocytes
*TTLL7*	black	Unknown	Cardiac fibroblasts
*TXNDC5*	magenta	ECM/Cell adhesion, Fibrosis, ER processes, Oxidative stress	Not detected in experiments
*UGCG*	black	Energy metabolism, Contractility	Endothelial cells
*WARS1*	magenta	Angiogenesis, Apoptosis	Endothelial cells

The module is the module assignment from WGCNA. Pathways are putatively assigned based on GO enrichment terms and literature review as detailed in Supplementary Table 10. ‘ER processes’ includes protein folding and protein synthesis, while ‘ECG’ refers to changes in intervals measured by electrocardiogram. ECG = Electrocardiography, ECM = Extracellular Matrix, ER = Endoplasmic Reticulum.

We also tested whether the 5 MEs could be used to predict SCT. However, MEs could not differentiate between structural and non-structural cardiotoxins (Supplementary Figure 8A). Only a random forest classifier using four uncorrelated MEs as features could distinguish between structural and non-structural cardiotoxins. This model performed poorly in cross-validation compared to the dummy classifier (Supplementary Figure 8B).

## Discussion

SCT presents a high-impact risk that is difficult to assess in early drug discovery. By applying machine learning methods to phenotypic and transcriptomic data in physiologically-relevant cardiac models across multiple cardiotoxic and non-cardiotoxic compounds, we identified additional phenotypic assay parameters and molecular markers which in concert capture diverse mechanisms of SCT.

In this study, we identified nine calcium transient parameters which were associated with SCT. Among them was peak count, which is commonly used to assess functional cardiotoxicity (Pointon et al. 2015). The other eight parameters comprised of intensity, mean peak width at 10% and 25% prominence, and average measures of the shoulder, tail, and valley durations of a peak. While these parameters performed better than peak count and average amplitude alone, we observed modest overall performance and large variations in model performance during cross-validation. This is likely due to the heterogeneous profile of structural cardiotoxins, where severe structural cardiotoxins may or may not act as inotropes (Pointon et al. 2013). A recent study also used CardioWave to derive 40 calcium transient parameters and build a random forest classifier of 48 inotropes and non-inotropes (Yang et al. 2023). The random forest model built in the previous study had a high AUC (0.90 – 0.94), though its specificity was lower (0.57 – 0.64) and only 2 of the 13 important features were shared with this study (mean peak width (10%) and mean peak width (25%) (Yang et al. 2023). Differences in model performance and parameters identified may be explained by the different model endpoints used. While calcium transients alone are unlikely to fully explain mechanisms of SCT, they strongly account for mechanisms of inotropy (Lee and Allen 1997). Indeed, other organelle-level assays like the HCB assay have been proven to be more predictive for SCT (Archer et al. 2018). We did not compare the predictivity of HCB-derived parameters with our calcium transient parameters in this study, however, future work may be performed to test whether parameters from these assays may be used in complement to gain a comprehensive assessment of SCT.

We also characterised the transcriptomic profiles of structural and non-structural cardiotoxins. The number of genes affected by structural cardiotoxins tended to increase with time and concentration; however, doxorubicin had a low impact on gene expression profiles (Supplementary Table 3). This could be due to doxorubicin’s mechanism of action as an inhibitor of topoisomerase 2A, which is required for transcription (Hortobágyi 1997). We identified 52 genes which were expressed in one or more cardiac cell types that captured diverse mechanisms of SCT induced by one or more compounds. Based on existing literature, these genes were putatively mapped to biological mechanisms with plausible links to SCT, including extracellular matrix organisation and cell adhesion, oxidative stress, fibrosis, inflammation, apoptosis, and calcium signalling (Supplementary Table 10).

Calcium homeostasis plays an important role in cardiac contractility (Connell et al. 2020; McGregor et al. 2004) and is known to be affected by compound-induced cardiotoxicity (Pointon et al. 2013, 2015; Yang et al. 2022; Yuan et al. 2020). In this study, five genes (*NPY1R*, *NPY2R*, *RNF24*, *RPS6KA3*, and *UGCG*) were putatively linked to mechanisms of contractility. *NPY1R* and *NPY2R*, which regulate calcium channel abundance and cardiac left ventricle morphogenesis (Jacques et al. 2003; Safran et al. 2021), have been associated with left ventricular hypertrophy (Arnett et al. 2009). Of the other three genes, *RNF24* is an integral membrane protein which regulates calcium ion influx (Safran et al. 2021), *RPS6KA3* encodes a kinase which increases the beat amplitude of cardiomyocytes (Lamore et al. 2017), and *UGCG* is associated with contractility response to a known cardiotoxin, dobutamine (Andersson et al. 2021). In this study, *NPY1R* and *NPY2R* were significantly up-regulated while *RNF24*, *RPS6KA3*, and *UGCG* were significantly down-regulated in structural cardiotoxins compared to non-structural cardiotoxins (Supplementary Table 10). These observed trends are in accordance with cardioprotective response to doxorubicin in mice (Eder and Molkentin 2011; Mattila et al. 2020; Norton et al. 2022) and indicate that they are responses to compound-induced SCT.

In heart failure, there is a shift in cardiomyocytes away from fatty acid beta-oxidation to glucose oxidation (Doenst et al. 2013). This shift has been detected in proteomic studies of compound-induced cardiotoxicity (Brandão et al. 2022), although directions of effect on proteins related to glycolysis and lipid metabolism depend heavily on the compound in question (Brandão et al. 2022). Among the 52 genes prioritised in this study, 6 (*ASXL3*, *EPHX1*, *FAM13C*, *LPCAT1*, *PLSCR4*, and *UGCG*) were linked to lipid metabolism, 1 (*PGD)* was linked to the pentose phosphate pathway, and 1 (*SLC1A5*) was linked to glutamine homeostasis (Supplementary Table 10). Among the lipid metabolism-associated genes, only *ASXL3* and *FAM13C* were up-regulated in structural cardiotoxins compared to non-structural cardiotoxins, though this is expected as *ASXL3* negatively regulates lipogenesis (Shin et al. 2014). In contrast, *PGD* and *SLC1A5* were down-regulated in structural cardiotoxins compared to non-structural cardiotoxins (Supplementary Table 5). These results indicate a greater reliance of cardiac cells on glycolysis and reduced flux through the lipid metabolism and pentose phosphate pathways (Cho et al. 2018).

Transcriptomics datasets generated using small sample sizes and bespoke study designs are often noisy and highly variable; therefore, bioinformatics methods applied to the same dataset may yield different results (Baik et al. 2020; Sánchez-Baizán et al. 2022). In this study, we therefore used complementary methods employing different assumptions and approaches to yield a list of high-confidence genes linked to SCT. Differential expression analysis models the variance and mean counts across genes to identify genes which significantly change in expression between conditions (Love et al. 2014). While this approach robustly modelled wide variations in expression across genes and provides intuitive hypothesis testing and interpretable results, it also suffered from small sample sizes which could introduce large variation or outliers and high uncertainty in estimates. By contrast, co-expression network approaches like WGCNA constructs modules of genes which are co-expressed both at the gene level and the network topology level (Langfelder and Horvath 2008), with hub genes acting as highly-connected and representative nodes within the modules. While WGCNA therefore enables more robust functional annotation and correlation with traits of interest, it is still susceptible to small sample sizes, and identified hub genes may only represent genes correlated to the true mediating genes of SCT effects. Among the 17 protein-coding genes prioritised in differential expression analysis and included in WGCNA, 11 were members of SCT-correlated modules, thus indicating the value of employing orthogonal methods to prioritise genes.

Machine learning has recently gained traction in biomedical research as a method to identify important predictors of cardiotoxicity and cardiovascular disease (DeGroat et al. 2024; Grafton et al. 2021; Qian et al. 2023), though these methods are not without risks. Given our small dataset of twelve compounds, we used simple and more interpretable machine learning methods to identify 52 genes which could be potential biomarkers to assess and de-risk SCT. While we performed compound-specific cross-validation and fine-tuned the hyperparameters of each model to optimise model performance, the small size of the dataset our machine learning models were training on precluded the use of an independent test set. Therefore, our machine learning models may be overfit, i.e. demonstrate poor generalisability to other transcriptomics datasets. Validation presents an useful way to test the generalisability and utility of the 52 prioritised genes for SCT classification, however, at the time of writing we were not aware of other transcriptomics datasets derived from a comparable human-derived, *in vitro* cell model exposed to a diverse set of structural and non-structural cardiotoxins. As the goal of this work was to identify a putative shortlist of genes linked to SCT, future validation in independent transcriptomic studies is required to increase confidence for use in decision making during drug discovery.

We previously mentioned that existing SCT assays lack mechanistic interpretability beyond the organelle level. WGCNA analysis provided insight into observed trends of SCT-associated modules with relation to assay parameters, namely, that HCB parameters were correlated with module eigengenes in the same direction as SCT while calcium transient parameters were correlated in the opposite direction. We also identified module eigengenes which were significantly correlated with HCB parameters and/or calcium transient parameters but not with SCT, demonstrating that these parameters may capture other compound-specific effects which could lend noise to the dataset. Although this was beyond the scope of our study, future work to understand the correlation between variations in these parameters with gene expression signatures will be valuable for improving the specificity of SCT assessment assays.

Core strengths of the study include the range of structural and non-structural cardiotoxins assessed, which allowed us to identify novel genes in pathways associated with SCT, and the use of multi-cell type, human cardiac *in vitro* models, as we identified several genes which were expressed in endothelial cells and/or fibroblasts, but not in cardiomyocytes (Supplementary Tables 5^1^, 10). However, these strengths also presented some limitations. One limitation of this study was the hiPS-CMs we used. In particular, our hiPS-CMs presented a relatively immature phenotype which could have altered the calcium transient and transcriptomic findings we report. Nevertheless, these hiPS-CMs have previously been shown to capture transcriptomic changes linked to cardiotoxicity (Chaudhari et al. 2016; Matsa et al. 2016; McSweeney et al. 2019), and in the case of the calcium transients, present a contractile phenotype which more mature models like the PromoCell GmbH (Heidelberg, Germany) cardiomyocytes lack (van Hasselt et al. 2020, https://promocell.com/product/human-cardiac-myocytes-hcm/#tab-description). Finally, while the assigned pathways demonstrate putative functions of the 52 prioritised genes in SCT, we lacked the resolution to link pathways to specific cell types or identify biological pathways which might contribute more to SCT than others. Following validation of our genes, we highlight single-cell or spatial transcriptomics as one avenue of follow-up, as such studies have already successfully linked gene changes to individual cell types (Kanemaru et al. 2023; Walls et al. 2023).

Here, we showed that phenotypic assays applied to *in vitro* model systems can be used *in tandem* to capture the multi-faceted mechanisms of SCT. We also show that bioinformatic and machine learning approaches can identify a transcriptomic fingerprint of SCT which may be used to de-risk compounds in early drug discovery. Future work may expand upon these findings by increasing the number of structural cardiotoxins studied, integrating other omics technologies to increase confidence in these findings, and assessing the genes highlighted here as potential markers in targeted assays of SCT.

## Experimental Procedures

Details about cell culture, RNA-seq library preparation and sequencing, and data acquisition using the calcium transient and high content biology assays can be found in the Supplementary Experimental Procedures.

### Compound selection and annotation

A total of 46 clinical compounds (48% structural cardiotoxins) representing chemically-diverse structural and non-structural cardiotoxins were included for calcium transient analysis (Supplementary Table 2). Compounds were considered structural cardiotoxins if the corresponding FDA label referenced heart failure or a decrease in left ventricular ejection fraction. Other compounds were labelled as non-structural cardiotoxins, even if the corresponding FDA label referenced non SCT-related cardiac pathologies.

A more selective approach was performed to select compounds for transcriptomic profiling. Eight structural cardiotoxins which represented a range of chemical classes contributing to SCT and which had in-house data available for the HCB assay were selected. In addition, four chemically-diverse non-structural cardiotoxins which were correctly predicted in the HCB assay were chosen to enable clear separation of structural cardiotoxins from non-structural cardiotoxins and represent a full diversity of responses.

### Feature selection for calcium transient parameters associated with SCT

For feature selection, waveform parameters which were uncorrelated (Pearson R^2^ < 0.95) and had high variance (ơ^2^ > 0.02) were used to build classification models of SCT. Ten times repeated, five-fold cross-validated models of SCT were built on 46 clinical compounds (48% structural cardiotoxins) with the Python scikit-learn v1.0.2 package (Pedregosa et al. 2011). To select the best models for selecting features, seven models were compared: logistic regression, elastic net, ridge regression, random forest, support vector machine, K nearest neighbours, and Gaussian (Naïve Bayes) models. A randomised search grid algorithm was used to tune and select hyperparameters for each model. Area under the curve (AUC), precision, and recall were assessed, and models were tested on the 46 clinical compounds (48% structural cardiotoxins) used to train the model. Model performances were compared to performance of a dummy classifier which makes stratified predictions using the Python scikit-learn v1.0.2 package (Pedregosa et al. 2011). Stratified prediction accounts for potential differences in cross-fold validation performance by accounting for class imbalance in the dataset (Pedregosa et al. 2011).

The ten features with the largest absolute weightings were obtained for ridge regression and elastic net, and the ten features with the largest mean decrease in GINI were obtained for random forest. Features were considered as associated with SCT if they were selected in at least two of these selected models. To test whether selected features could discriminate between structural cardiotoxins and non-structural cardiotoxins, a logistic regression classifier of selected features was compared with a baseline classifier containing only peak count and average amplitude as features.

### Transcriptomic profiling

In short, transcriptomic profiles were generated from cardiac microtissues by sequencing mRNA-seq libraries on an Illumina sequencing platform.

Cardiac microtissues, which were cultured and prepared for sequencing as detailed in the Supplementary Experimental Procedures, were exposed to 12 compounds (8 structural cardiotoxins, 4 non-structural cardiotoxins) at two concentrations (low and high) and three timepoints (6, 48, and 72 hrs) with three biological replicates and one technical replicate. High concentrations were determined for each compound based on the lowest concentration of either i) IC_30_ of ATP depletion (i.e. cell death) in cardiac microtissues in a high content biology assay associated with SCT; ii) ≤15x total C_max_ (to maintain therapeutic relevance); or iii) lowest investigated concentration (Archer et al. 2018). The half-log dilution of each high concentration was then used as the low concentration. ATP depletion was not seen in cardiac microtissues for any of the compounds at the low concentration. A summary of the compounds, annotations, and concentrations tested can be found in Supplementary Table 2.

### Differential gene expression analysis

Raw count data was batch-corrected using ComBat-seq in the R package sva v3.40.0(Zhang et al. 2020), then normalised and variance stabilisation transformed (vst-transformed) using the R package DESeq2 v1.32.0 (Love et al. 2014).

To characterise the gene expression changes induced by individual compounds, compound samples at each treatment were compared to batch- and timepoint-matched 0.1% (v/v) DMSO vehicle control samples using DESeq2 v1.32.0. Hierarchical clustering of treatments with the largest number of significant differentially expressed genes for each compound (abs(log_2_(Fold-Change) > 1, FDR *p*-value ≤ 0.05) was performed to identify similar gene expression profiles across compounds, and over-representation analysis of clustering gene expression profiles was performed using Ingenuity Pathway Analysis v90348151 (Qiagen Inc., https://www.qiagenbioinformatics.com/products/ingenuitypathway-analysis) (Krämer et al. 2014) (abs(activation Z-score) ≥ 2, FDR *p*-value ≤ 0.05). Differential expression analysis was also performed using DESeq2 v1.32.0 (Love et al. 2014) to characterise the gene expression changes in structural cardiotoxins compared to non-structural cardiotoxins, and over-representation analysis of significant genes was performed using the R package clusterProfiler v4.0.5 (Wu et al. 2021).

We also compared the gene expression profiles of structural cardiotoxins to non-structural cardiotoxins in an independent transcriptomic dataset comprising of 342 samples across 21 kinase inhibitors (5 structural cardiotoxins, 16 non-structural cardiotoxins). Samples were obtained from adult human cardiomyocytes from four healthy volunteers (two male, two female) that were exposed to kinase inhibitors at maximal concentrations for 48 hours (van Hasselt et al. 2020). Cell line-adjusted, normalised, and vst-transformed gene counts were compared between structural cardiotoxins and non-structural cardiotoxins using DESeq2 v1.32.0 (Love et al. 2014). Significance was assessed at an FDR-adjusted *p*-value ≤ 0.05.

### Weighted gene correlation network analysis

Weighted gene correlation network analysis (WGCNA) (Langfelder and Horvath 2008) was performed on batch-corrected, normalised, and vst-transformed gene counts from structural cardiotoxin and non-structural cardiotoxin samples at 48 hrs and 72 hrs. Spearman’s correlations were calculated for 33,778 genes with Ensembl gene IDs to create a signed network. After setting the seed to 42, a soft thresholding power of 5 was determined using the ‘pickSoftThreshold’ command, as it was the minimum value which exceeded an independence degree of 0.80 (Supplementary Figure 9). Distinct clusters were identified based on hierarchical clustering using Euclidean distance metrics with a distance threshold ≥ 0.25. MEs were tested for significant correlation (adjusted *p* ≤ 5x10^-3^) with the following:

1. FDA labels of SCT
2. pIC50 values of ATP depletion, ER integrity, and MMP as measured by the HCB imaging assay (Archer et al. 2018)
3. pIC50 values of calcium transient assay parameters which were associated with SCT in this study

Important genes were identified either by identifying important module members which i) had expression profiles correlated with SCT (*p* ≤ 1.48x10^-6^, adjusting for 33,778 genes), or ii) were among the top 5% most connected genes within a module as measured by intramodular connectivity.

To determine whether the inclusion of vehicle controls affected gene clustering, additional WGCNA networks were constructed. These networks included i) all vehicle control, structural cardiotoxin, and non-structural cardiotoxin samples at 48 hrs and 72 hrs, and ii) vehicle control and structural cardiotoxin samples at 48 hrs and 72 hrs. To assess model robustness, MEs were correlated between networks.

WGCNA was performed using the R package WGCNA v1.72-1 (Langfelder and Horvath 2008).

### Machine learning models of SCT using prioritised genes and MEs

Classifiers of SCT were built using the combined set of genes prioritised in differential expression and WGCNA analysis in scikit-learn 1.0.2 (Pedregosa et al. 2011). Batch-corrected and normalised counts of genes which were uncorrelated (Pearson R^2^ < 0.95) and had high variance (ơ^2^ ≥ 0.02) were included as features, and a standard scaler was applied to normalise values across the dataset. Samples at all concentrations after 6 hours were used (*n*=144, 67% structural cardiotoxins). The dataset was split into 80% training and 20% test sets, ensuring that all samples belonging to the same compound were only in the training or only in the test set, and that class balance reflected that in the full dataset after splitting. Hyperparameters to build the logistic regression, elastic net, and random forest classifiers were tuned using a randomised search grid algorithm, and three-times repeated, stratified four-fold cross-validation was performed on a per-compound basis to obtain robust results. Classifier models were compared for performance based on AUC, precision, and recall with performance from a dummy classifier built using stratified predictions in scikit-learn v1.0.2 (Pedregosa et al. 2011) to account for class imbalance. Assigned weights were used to identify important features in logistic regression and elastic net classifiers, and mean GINI decrease was used for the random forest classifier. Genes with absolute weight values in the top 90^th^ percentile were prioritised for each model.

Classifiers of SCT were also built and assessed using MEs associated with SCT as features. The analysis performed was largely similar to that for the gene-feature classifiers except that MEs were not filtered by variance, and significant features were assessed using weight values in the top 80^th^ percentile.

### Annotation of prioritised genes

Prioritised genes from machine learning models were annotated for function based on multiple sources of evidence: i) gene ontology (GO) terms based on over-representation analysis of WGCNA modules performed with clusterProfiler v4.0.5(Wu et al. 2021); ii) gene function, as reported in GeneCards (Safran et al. 2021); iii) differential expression analysis of structural cardiotoxins compared to non-structural cardiotoxins in this study, cross-referenced against results from individual compounds compared to timepoint- and batch-matched controls; iv) tissue heart expression from the Gene-Tissue Expression Atlas consortium version 8 (GTEx v8.0) (Aguet et al. 2020), and v) single cell expression in cardiomyocytes, endothelial cells, and cardiac fibroblasts obtained from clusters c-2, c-6, and c-7 in the Human Protein Atlas (Karlsson et al. 2021), respectively, as queried on May 25, 2023. A literature review of the 52 genes was also performed to assess whether they were previously identified in omics studies of cardiac development or cardiotoxicity. After assembling this evidence, pathways were assigned to genes in an expansive manner based on observations.

### Software

All analyses and graphics were performed and generated using R 4.1.0 (R Core Team 2021) and Python 3.8.12 (Van Rossum and Drake 2009).

## Supplementary information

ESM 1 (XLSX 1.62 mb)

ESM 2 (DOCX 1.87 mb)

ESM 3 (DOCX 99.8 kb)

## Data Availability

The data underlying this manuscript is available on reasonable request.

## Code availability

The code to perform analyses is available at https://github.com/vauyeung38/sct-transcriptomics-waveforms.
