# Supplementary material for: Computational approaches identify a transcriptomic fingerprint of drug-induced structural cardiotoxicity
Source: Cell Biol Toxicol. 2024 Jun 28;40(1):50. doi: 10.1007/s10565-024-09880-7 (PMC11213733; doi:10.1007/s10565-024-09880-7)

**Computational approaches identify a transcriptomic fingerprint of drug-induced structural cardiotoxicity**

**Submission to Journal: Cell Biology and Toxicology**

Victoria P.W. Au Yeung^1,2^, Olga Obrezanova^3^, Jiarui Zhou^4^, Hongbin Yang^5^, Tara J. Bowen^4^, Delyan Ivanov^6^, Izzy Saffadi^1^, Alfie S. Carter^1^, Vigneshwari Subramanian^7^, Inken Dillmann^8^, Andrew Hall^1^, Adam Corrigan^2^, Mark R. Viant^4,9^, Amy Pointon^1^

^1^Safety Sciences, Clinical Pharmacology & Safety Sciences, R&D, AstraZeneca, Cambridge, UK

^2^Phenomics, Data Sciences & Quantitative Biology, R&D AstraZeneca, Cambridge, UK

^3^Imaging and Data Analytics, Clinical Pharmacology & Safety Sciences, R&D, AstraZeneca, Cambridge, UK

^4^School of Biosciences, University of Birmingham, Edgbaston, Birmingham, UK

^5^Centre for Molecular Informatics, Department of Chemistry, University of Cambridge, UK

^6^High-Throughput Screening, R&D, AstraZeneca, Alderley Park, UK

^7^Imaging and Data Analytics, Clinical Pharmacology & Safety Sciences, R&D, AstraZeneca, Gothenburg, Sweden

^8^Disease Molecular Profiling, Discovery Biology, R&D AstraZeneca, Gothenburg, Sweden

^9^Phenome Centre Birmingham, University of Birmingham, Edgbaston, Birmingham, UK

Contact Information

Victoria P.W. Au Yeung: [victoria.auyeung.publications@gmail.com](mailto:victoria.auyeung.publications@gmail.com); ORCID ID: 0000-0002-0823-3963

**Supplementary Figures**

**Figure 1:** **Machine learning results for seven machine learning models tested using 17 calcium transient parameters as features and SCT label as an outcome.** Triangles represent performance on the full compound set used to train each classifier. Model performances are compared to those of a dummy classifier which predicts SCT accounting for the slight class imbalance in the 46 compounds (48% structural cardiotoxins). Dummy = dummy classifier with stratified predictions; Eln = Elastic net regression; GNB = Gaussian (Naïve Bayes); KNN = K-nearest neighbours; Logregcv = Logistic regression; RF = Random forest; Ridge = Ridge regression; SVM = Support vector machine.

**Figure 2:** **Principal components analysis of 500 genes with the greatest variation in gene expression profiles of cardiac microtissues exposed to one of 8 structural cardiotoxins and 4 non-structural cardiotoxins across two concentrations and three timepoints.** Plots are coloured by **A)** batch, and controls within batch; **B)** compound; **C)** timepoint, and controls within timepoint; **D)** concentration, and **E)** SCT.

**Figure 3: Hierarchical clustering of 4,292 genes which are significantly associated (FDR-adjusted p-value ≤ 0.05) with at least one of the twelve compound treatments.** Compounds are grouped into four clusters based on the horizontal blue line across the hierarchical clustering tree.

**Figure 4: PLS-DA analysis replicates multiple genes associated with SCT in differential gene expression analysis. A)** PLS-DA parameter tuning. The classification error rate is lowest with 2 principal components, so PLS-DA was performed using 2 principal components. **B)** PLS-DA scores show good discrimination between structural cardiotoxins and non-structural cardiotoxins. **C)** Rank distribution of 2,649 differentially expressed genes (in structural cardiotoxins compared to non-structural cardiotoxins analysis, FDR-adjusted p ≤ 0.05) compared to 11,710 non-significant genes. The lower the rank number, the larger the absolute weight of the gene in PLS-DA.

**Figure 5: ME correlations between modules identified in A) structural cardiotoxins and non-structural cardiotoxins network compared to a network with DMSO, structural cardiotoxins and non-structural cardiotoxins samples, and B) the structural cardiotoxins and non-structural cardiotoxins network compared to a DMSO and structural cardiotoxins network.** Only cells with a significant p-value (Bonferroni p ≤ 0.05 * number of modules in network 1 * number of modules in network 2) are labelled with the Pearson’s R^2^ correlation and p-value in brackets. Module labels annotated in black are those associated with SCT (p ≤ 5x10^-3^). Structural cardiotoxins are abbreviated to ‘Toxins’ and non-structural cardiotoxins to ‘Non-toxins’.

**Figure 6: Gene significance of SCT plotted by module membership for (A) magenta, B) green, (C) darkgrey, (D) orange, and (E) black modules.** Pearson’s R^2^ correlation and p-value associations between gene significance and module membership are shown. Genes which were prioritised in differential expression analysis of SCT are labelled. Orange boxes mark the areas in which genes were significantly associated with structural cardiotoxins compared to non-structural cardiotoxins (Bonferroni p ≤ 1.48x10^-6^) and had strong module membership (Bonferroni p ≤ 1.48x10^-6^).

**Figure 7: Gene significance of SCT plotted by intramodular connectivity for (A) magenta, B) green, (C) darkgrey, (D) orange, and (E) black modules.** Pearson’s R^2^ correlation and p-value associations between gene significance and module membership are shown. Genes which were prioritised in differential expression analysis of SCT are labelled. Orange boxes mark the areas in which genes were significantly associated with structural cardiotoxins compared to non-structural cardiotoxins (Bonferroni p ≤ 1.48x10^-6^) and were in the top 95^th^ percentile of connectivity.

**Figure 8: Discrimination of structural and non-structural cardiotoxins by MEs. A)** Principal component analysis of 5 MEs on structural cardiotoxins and non-structural cardiotoxins. ME loadings are labelled. **B)** Performance of a random forest machine learning model with 4 uncorrelated (Pearson R^2^ < 0.95) MEs as features and FDA label of SCT as the outcome compared to a dummy classifier. Box-and-whisker plots denote performance from 3 times repeated 4-fold cross-validation, yellow points represent overall AUC, red points represent performance for structural cardiotoxins, and blue points represent performance for non-structural cardiotoxins in the full dataset.

**Figure 9:** **Parameter tuning from WGCNA.** A) Scale independence as a function of soft threshold power. B) Mean connectivity as a function of soft threshold power.

**Figure 1**


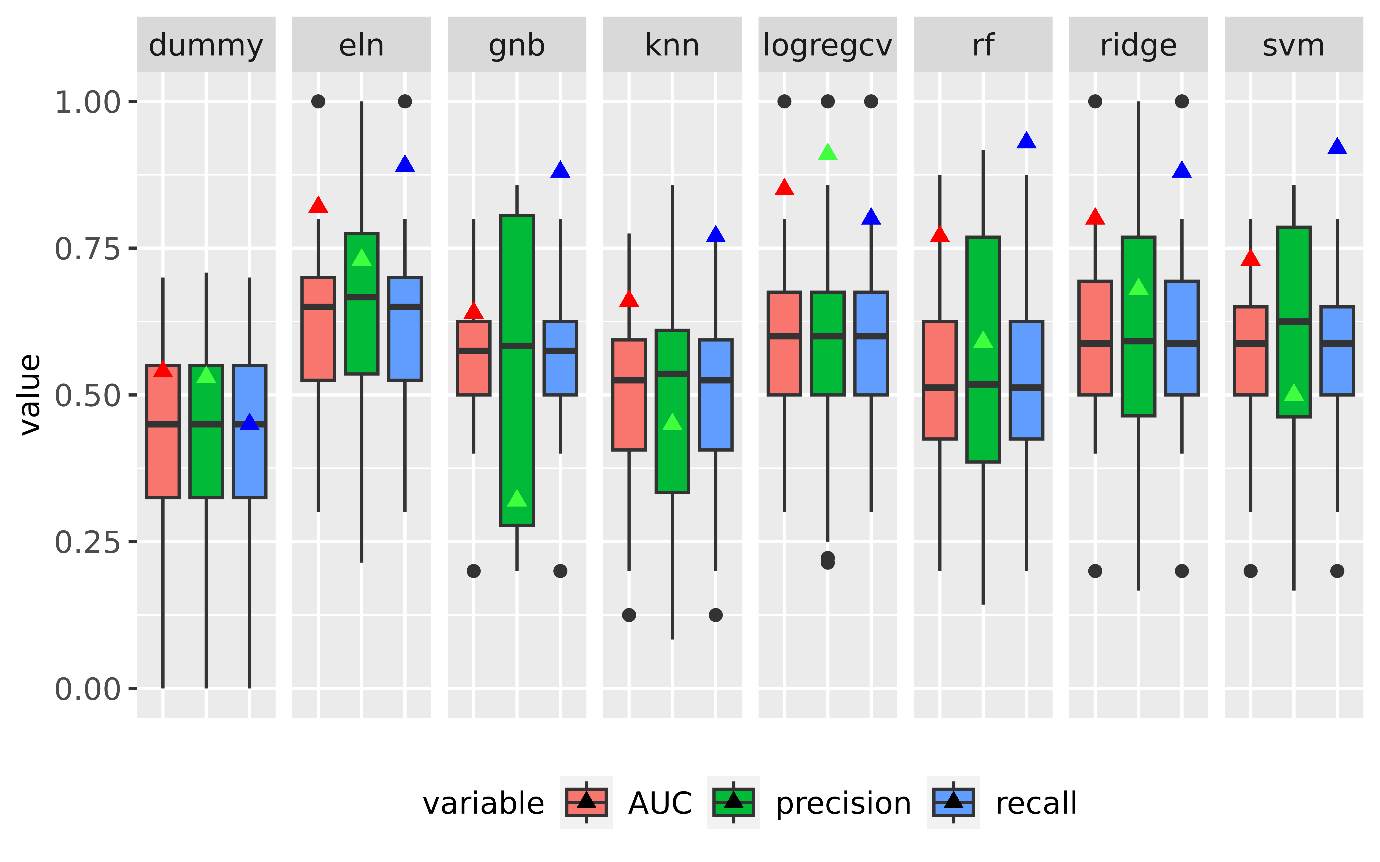


**Figure 2**


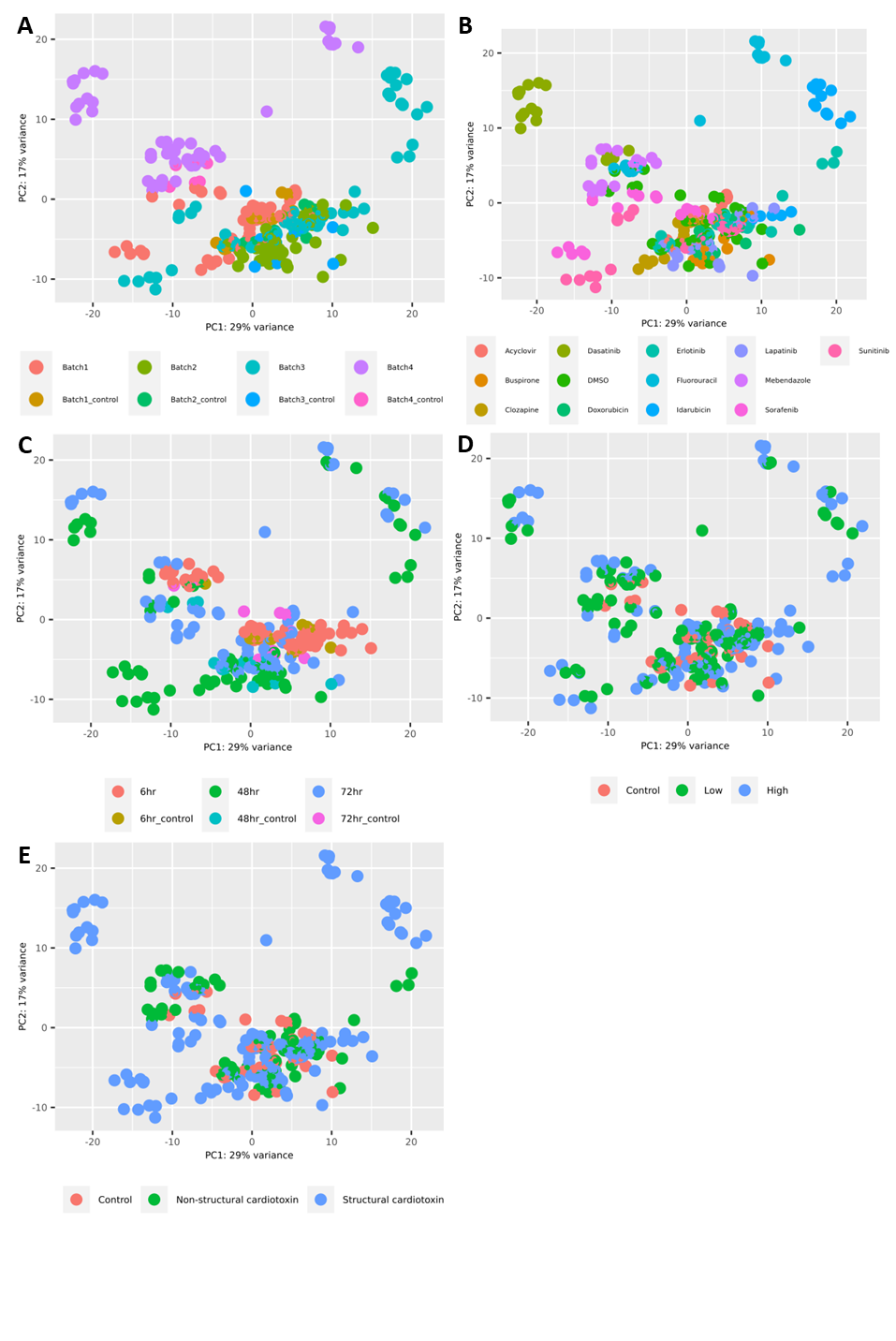


**Figure 3**

**
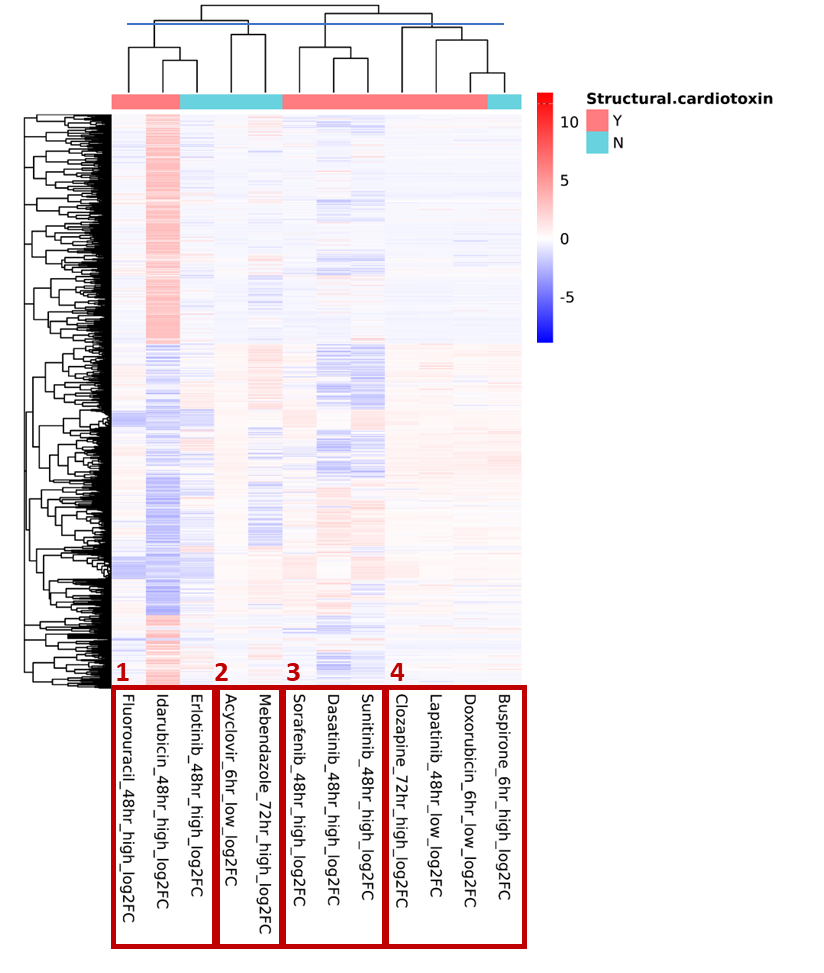
**

**Figure 4**


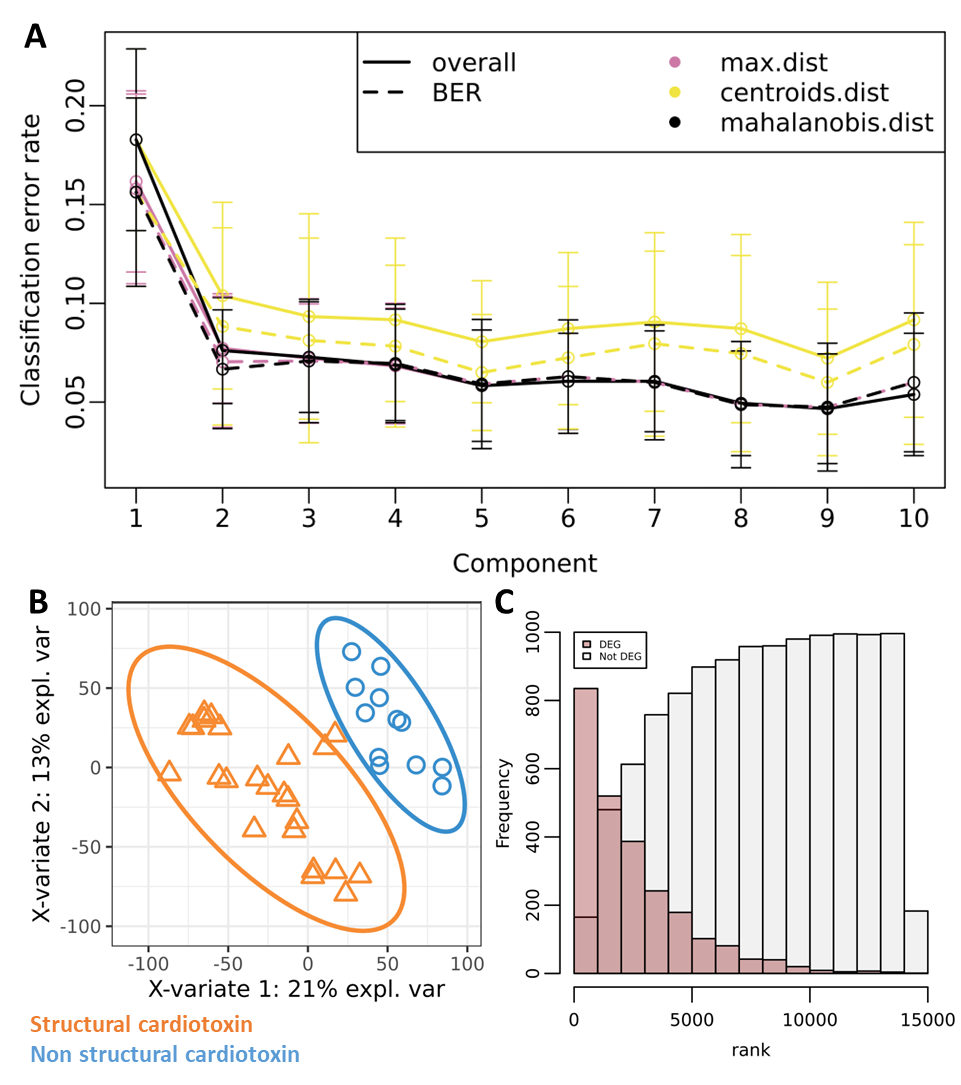


**Figure 5**


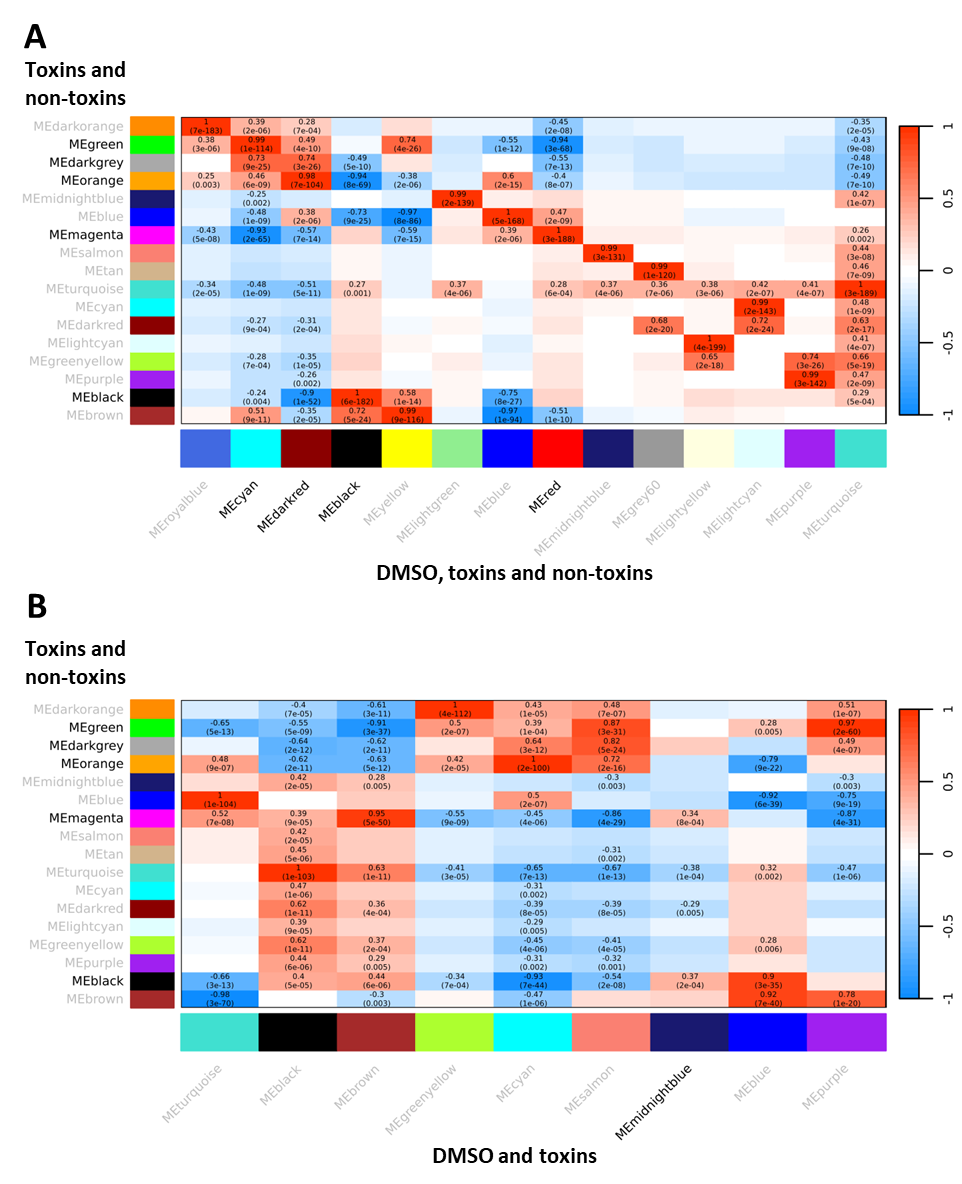


**Figure 6**


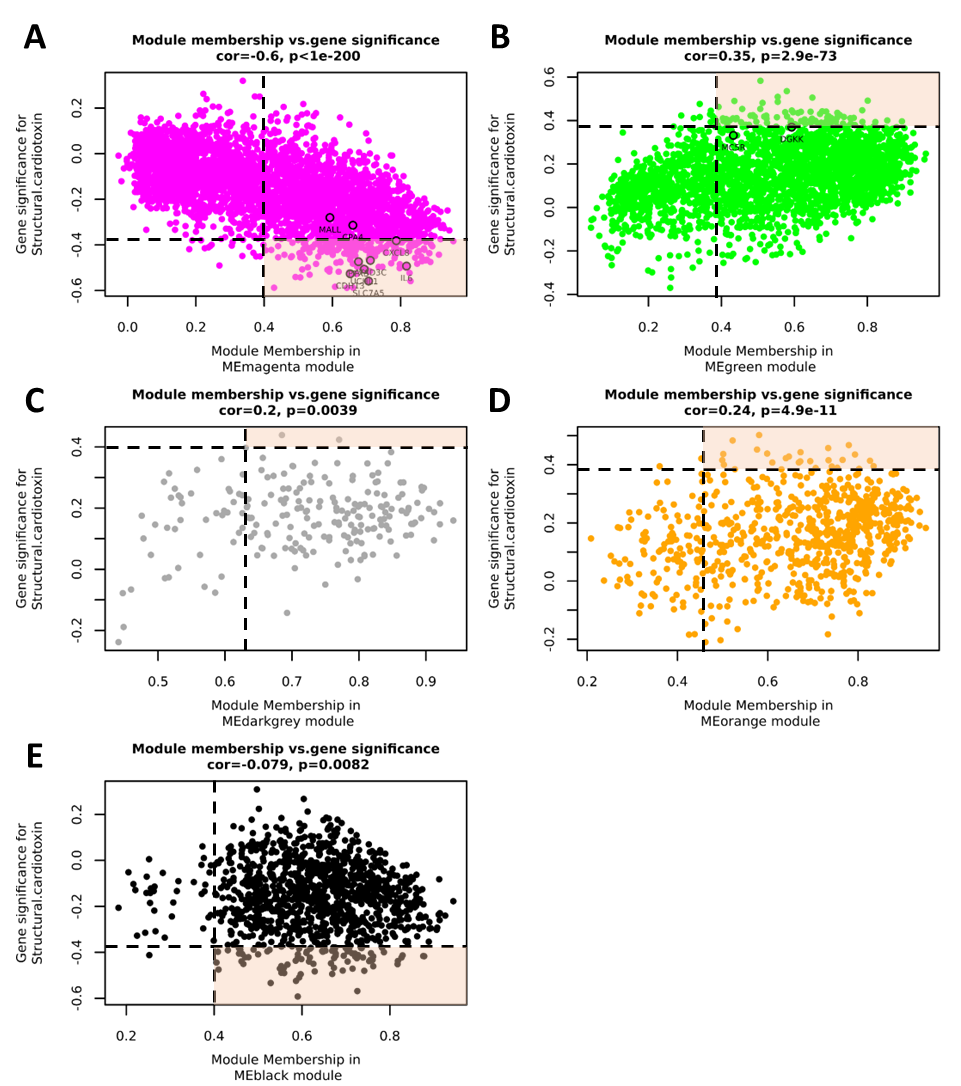


**Figure 7**


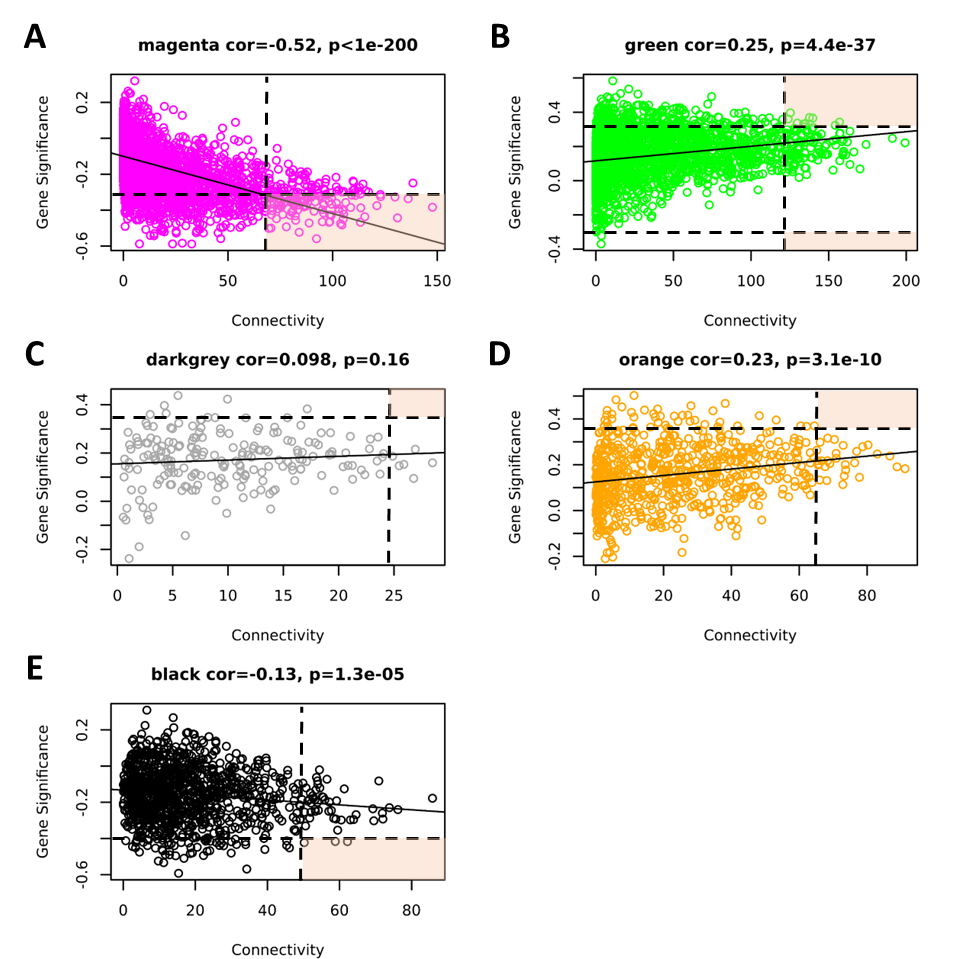


**Figure 8**


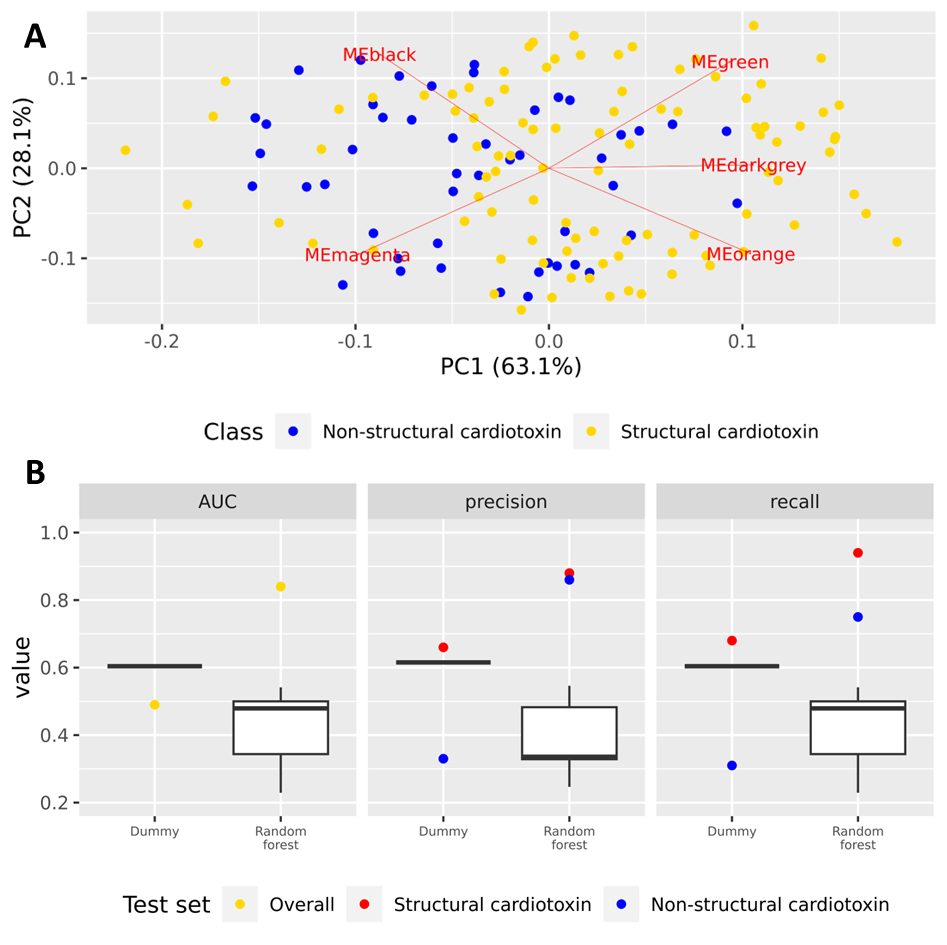


**Figure 9**


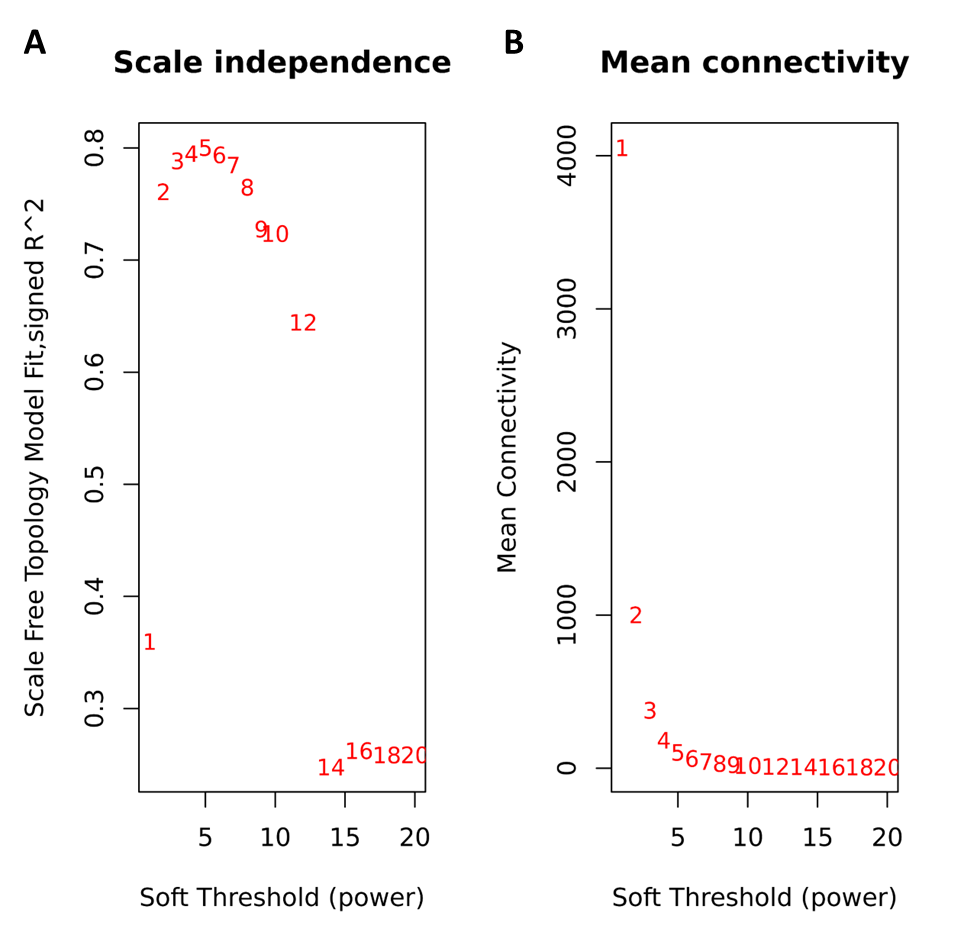

Supplement: Supplementary file 2 — (DOCX 1.87 mb) [file 10565_2024_9880_MOESM2_ESM.docx]
