# Supplementary material for: Computational approaches identify a transcriptomic fingerprint of drug-induced structural cardiotoxicity
Source: Cell Biol Toxicol. 2024 Jun 28;40(1):50. doi: 10.1007/s10565-024-09880-7 (PMC11213733; doi:10.1007/s10565-024-09880-7)
Supplement: Supplementary file 3 — (DOCX 99.8 kb) [file 10565_2024_9880_MOESM3_ESM.docx]

**Computational approaches identify a transcriptomic fingerprint of drug-induced structural cardiotoxicity**

**Submission to Journal: Cell Biology and Toxicology**

Victoria P.W. Au Yeung^1,2^, Olga Obrezanova^3^, Jiarui Zhou^4^, Hongbin Yang^5^, Tara J. Bowen^4^, Delyan Ivanov^6^, Izzy Saffadi^1^, Alfie S. Carter^1^, Vigneshwari Subramanian^7^, Inken Dillmann^8^, Andrew Hall^1^, Adam Corrigan^2^, Mark R. Viant^4,9^, Amy Pointon^1^

^1^Safety Sciences, Clinical Pharmacology & Safety Sciences, R&D, AstraZeneca, Cambridge, UK

^2^Phenomics, Data Sciences & Quantitative Biology, R&D AstraZeneca, Cambridge, UK

^3^Imaging and Data Analytics, Clinical Pharmacology & Safety Sciences, R&D, AstraZeneca, Cambridge, UK

^4^School of Biosciences, University of Birmingham, Edgbaston, Birmingham, UK

^5^Centre for Molecular Informatics, Department of Chemistry, University of Cambridge, UK

^6^High-Throughput Screening, R&D, AstraZeneca, Alderley Park, UK

^7^Imaging and Data Analytics, Clinical Pharmacology & Safety Sciences, R&D, AstraZeneca, Gothenburg, Sweden

^8^Disease Molecular Profiling, Discovery Biology, R&D AstraZeneca, Gothenburg, Sweden

^9^Phenome Centre Birmingham, University of Birmingham, Edgbaston, Birmingham, UK

Contact Information

Victoria P.W. Au Yeung: [victoria.auyeung.publications@gmail.com](mailto:victoria.auyeung.publications@gmail.com); ORCID ID: 0000-0002-0823-3963

**Supplementary Experimental Procedures**

**Cell culture of hiPS-CMs and cardiac microtissues**

We acquired hiPS-CMs (iCell™ cardiomyocytes), thawing media, and maintenance media from Cellular Dynamics International (Madison, WI, USA). The hiPS-CMs were plated according to manufacturer’s instructions and as previously described (Pointon et al., 2015). Briefly, 384 well PhenoPlates (PerkinElmer) were coated with 0.1% gelatin. The hiPS-CMs were thawed and resuspended in 5 ml thawing media. Cells were pelleted at 300 g for five minutes before being resuspended in 10 ml thawing media and plated into the 384 well plate at a density of 5,000 cells per well in 50 µl. After 48 hours, the culture media was topped up to 100 µl with maintenance media. Cells were maintained using a maintenance media change every 48 hours until day 10 when cells were ready to use.

Cardiac microtissues were formed by combining hiPS-CMs with human cardiac microvascular endothelial cells (hCMECs) and primary human cardiac fibroblasts (hCFs) from Lonza (Basel, Switzerland). The hCMECs were cultured in endothelial basal medium 2 (Lonza, CC7030) while hCFs were cultured in fibroblast growth medium 3 (Lonza, CC2904) supplemented with growth factors (Archer et al., 2018).

The hCFs and hCMECs were detached using TrypLE (Thermo Fisher) while hiPS-CMs were thawed as described above. Microtissues were then formed as previously described (Archer et al., 2018) by mixing cell suspensions of hiPS-CMs, hCFs, and hCMECs to give a concentration of 500 cells per 40 µl in thawing microtissue media (50% iCell CM thawing media, 50% hCMEC media) to give a final cell ratio of 4 hiPS-CMs : 2 hCFs : 1 hCMECs. Cardiac microtissue media was topped up to 80 µl with maintenance microtissue media (50% iCell maintenance media, 50% endothelial basal medium 2) after 48 hours in culture. Microtissues were maintained by a 50/50 media change twice weekly until day 14 when microtissues were ready to use.

**Calcium transient data and feature derivation**

The calcium transient assay was performed as previously described (Pointon et al., 2013, 2015). Briefly, hiPS-CMs were cultured for 10 days, and iCell™ maintenance media was replaced every 48 hours leading up to the experiment. For the experiment, hiPS-CM cultures were exposed to compounds across a half-log 8 point concentration range starting at either 30 µM or 100 µM **(Supplementary Table 1)** for 72 hours in 3-5 technical replicates performed across 1-2 independent replicates. A fluorescent dye from the FLIPR Calcium Assay Kit (Molecular Devices) which stains for intracellular calcium ions was added (Sirenko et al., 2013) at 37^o^C for 30 minutes, along with 10 µM verapamil (positive control).

Cells were then transferred to a FLIPR Tetra, maintained at 37^o^C, and imaged. Fluorescence was evaluated at 480 nm excitation and 530 nm emission, and 800 reads of calcium flux were measured over 100 seconds. Raw data were also smoothed and scaled using a 5-point quadratic polynomial Savitzky-Golay filter (Savitzky & Golay, 1964) and by subtracting the minimum relative fluorescence unit (RFU) count from each well from every point in that well, as performed previously (Yang et al., 2022).

Following data measurement, calcium transient traces were plotted and assessed visually, and traces were excluded if traces had low amplitudes (<40 RFU), or if samples were 0.1% (v/v) DMSO vehicle controls which contained double peaks or irregular beat patterns.

To detect peaks, verapamil at 10 µM concentration (Harmer et al., 2012), which is known to inhibit almost all of the calcium transient signal in cardiomyocytes, was used to calculate a minimum peak detection threshold. The minimum peak detection threshold in this study was defined as the sum of the mean peak prominence and three times the standard deviation of peak prominence (Yang et al., 2022). The CardioWave v0.2.3 Python package (Yang et al., 2022) was used to identify 24 non-standard deviation related waveform parameters and normalise them to vehicle controls across plates. A full description of the derived parameters is provided elsewhere (Yang et al., 2022).

In CardioWave v0.2.3 (Yang et al., 2022), we used the ToxCast Pipeline Hill curve method to draw pIC_50_ curves across concentration ranges for each replicate. Aggregation of censored and uncensored pIC_50_ values across replicates for a given compound was performed using a maximum likelihood estimation method (McLoughlin et al., 2021) implemented in the Python ATOM Modeling Pipeline (AMPL) package (Minnich et al., 2020).

**Next-generation sequencing**

Fourteen microtissues per condition were pooled into one well of an Abgene Deepwell plate. The media was removed and microtissues lysed in 63 µl LBS buffer were supplemented with proteinase k (Beckman Coulter, California, USA). Samples were lysed at room temperature according to manufacturer’s (Beckman Coulter) guidelines and frozen at -80^o^C prior to RNA extraction.

Total RNA was extracted from thawed lysates using Agencourt RNAdvance Cell v2 kit (Beckman Coulter, A47943) on a Biomek i7 Hybrid system (Beckman Coulter) including Ambion DNAse I treatment (Thermo Fisher, AM2224) according to manufacturer’s instructions. RNA integrity and concentration were analysed on a Fragment Analyzer 5300 system using the RNA Standard Sensitivity kit (Agilent, DNF-471).

Libraries for mRNA-sequencing were prepared using the KAPA mRNA HyperPrep kit (Roche, KK8581) following manufacturer’s instructions with 75 ng total RNA input and 13 PCR cycles for library amplification. All library preparation steps were performed on a Tecan Fluent system (Tecan). Quality and concentration of the libraries were assessed on a Fragment Analyzer 5300 system using the NGS Standard Sensitivity kit (Agilent, DNF-473). The libraries obtained from each processing batch were pooled equimolarly, and bead washed at a 0.9x bead:sample ratio using HighPrep PCR clean-up beads (MagBio Genomics, AC-60500) to remove adapter dimers. The cleaned pools were quantified on the Qubit using the 1x dsDNA High Sensitivity kit (Thermo Fisher, Q33231) and pooled according to read ratios. The final pool was diluted and denatured according to Illumina’s recommendations and sequenced on the NovaSeq6000 system (Illumina) with paired-end 2x51bp configuration.

**Targeted qRT-PCR**

Targeted qRT-PCR was performed on a subset of significantly altered genes **(Supplementary Table 7)**. mRNA was extracted as detailed above and also extracted using the RNeasy kit (Qiagen Inc.). cDNA was synthesised using Vilo Superscript IV (Thermo Fisher), target gene expression was quantified using Taqman assay reagents according to manufacturer’s instructions, and data were analysed using Quant studio 6 (Invitrogen). The experiment was performed with three technical replicates across 1-2 independent replicates.

**High content biology (HCB) imaging assay**

The HCB imaging assay was performed as previously described (Archer et al., 2018). Briefly, cardiac microtissues were exposed to compound at a top test concentration of 158 µM or 50 µM with 9-point half-log dilutions for 72 hours, with four technical replicates. Cardiac microtissues were then stained with fluorescent probes by replacing 40 µl media from each well with 40 µl of 2x multiplexed dye for 30 minutes prior to imaging at 37^o^C, 5% CO_2_. ER-Tracker blue (2 µM) and TMRE (500 nM) were used as fluorescent probes to measure ER integrity and MMP, respectively. All microtissues were imaged on the Cell Voyager 7000 (Yokogawa Inc.), and images were captured on live plates using a 20x objective (Olympus UPLSAPO 0.75 NA, 0.6 mm WD) with a 2 x 2 bin. The ER-Tracker was imaged with a 405 nm excitation laser with a 445/45 nm band pass emission filter, while the TMRE was imaged using a 561 nm excitation laser and a 600/37 nm band pass emission filter. Images were captured over a 60 µm range in the Z-axis with a 5 µm interval between slices. This protocol was performed over multiple runs for each compound, and pIC_50_ values were aggregated using the maximum likelihood estimation method (McLoughlin et al., 2021) implemented in the Python ATOM Modeling Pipeline (AMPL) package (Minnich et al., 2020).

Cellular ATP concentrations were also assessed using the CellTiter Glo® Luminescent Cell Viability Assay as per the manufacturer’s instructions (Promega).

**Prioritisation of significant genes from differential gene expression analysis**

To identify genes robustly associated with structural cardiotoxicity, we first identified genes of large absolute effect size that were significantly differentially expressed (abs(log_2_(Fold-Change) > 1, FDR p-value ≤ 0.05) in structural cardiotoxins compared to non-structural cardiotoxins. Genes from this subset of differentially-expressed genes were prioritised if they were highlighted in one or more of the following approaches:

1. Replication in an independent transcriptomic profiling dataset: Differential expression analysis of structural cardiotoxins compared to non-structural cardiotoxins was performed using an independent transcriptomic dataset, as described above (van Hasselt et al., 2020). Significant genes at an FDR-adjusted p-value ≤ 0.05 with large absolute effect size (abs(log_2_(Fold-Change) > 1) which were found in both studies were prioritised.
2. Partial least squares determinant analysis (PLS-DA): To apply an approach that is less sensitive to low sample sizes, PLS-DA was performed using the R package mixOmics v6.16.3(Lê Cao et al., 2011) to discriminate between structural cardiotoxins and non-structural cardiotoxins and identify discriminating genes. The number of components used was selected by examining the base error rates of models permuted across a range of values (number of components: 1-10) using 3-fold cross-validation. Genes were prioritised if they were among the top 100 genes with the largest absolute weightings in PLS-DA.
3. Genes were also prioritised if they had absolute log2(Fold-Change) values ≥ 1.5 in the differential expression analysis performed in cardiac microtissues.

**Over-representation analysis**

To assess the function of modules identified in the WGCNA network of structural cardiotoxins and non-structural cardiotoxins, over-representation analysis was assessed. A total of 19,297 protein-coding genes identified in KEGG were included as background genes to test for over- or under-representation of KEGG GO terms. Significant associations were identified based on a Benjamini-Hochberg adjusted p-value ≤ 0.05. Over-representation analysis was performed using the R package clusterProfiler v4.0.5 (Wu et al., 2021).

**Supplementary References**

Archer, C. R., Sargeant, R., Basak, J., Pilling, J., Barnes, J. R., & Pointon, A. (2018). Characterization and Validation of a Human 3D Cardiac Microtissue for the Assessment of Changes in Cardiac Pathology. *Scientific Reports*, *8*(1), 1–15. https://doi.org/10.1038/s41598-018-28393-y

Harmer, A. R., Abi-Gerges, N., Morton, M. J., Pullen, G. F., Valentin, J. P., & Pollard, C. E. (2012). Validation of an in vitro contractility assay using canine ventricular myocytes. *Toxicology and Applied Pharmacology*, *260*(2), 162–172. https://doi.org/10.1016/j.taap.2012.02.007

Lê Cao, K. A., Boitard, S., & Besse, P. (2011). Sparse PLS discriminant analysis: Biologically relevant feature selection and graphical displays for multiclass problems. *BMC Bioinformatics*, *12*(1), 1–17. https://doi.org/10.1186/1471-2105-12-253/TABLES/3

McLoughlin, K. S., Jeong, C. G., Sweitzer, T. D., Minnich, A. J., Tse, M. J., Bennion, B. J., Allen, J. E., Calad-Thomson, S., Rush, T. S., & Brase, J. M. (2021). Machine Learning Models to Predict Inhibition of the Bile Salt Export Pump. *Journal of Chemical Information and Modeling*, *61*(2), 587–602. https://doi.org/10.1021/ACS.JCIM.0C00950

Minnich, A. J., Mcloughlin, K., Tse, M., Deng, J., Weber, A., Murad, N., Madej, B. D., Ramsundar, B., Rush, T., Calad-Thomson, S., Brase, J., & Allen, J. E. (2020). AMPL: A Data-Driven Modeling Pipeline for Drug Discovery. *Journal of Chemical Information and Modeling*, *60*(4), 1955–1968. https://doi.org/10.1021/ACS.JCIM.9B01053/SUPPL_FILE/CI9B01053_LIVESLIDES.MP4

Pointon, A., Abi-gerges, N., Cross, M. J., & Sidaway, J. E. (2013). Phenotypic profiling of structural cardiotoxins in vitro reveals dependency on multiple mechanisms of toxicity. *Toxicological Sciences*, *132*(2), 317–326. https://doi.org/10.1093/TOXSCI/KFT005

Pointon, A., Harmer, A. R., Dale, I. L., Abi-Gerges, N., Bowes, J., Pollard, C., & Garside, H. (2015). Assessment of Cardiomyocyte Contraction in Human-Induced Pluripotent Stem Cell-Derived Cardiomyocytes. *Toxicological Sciences*, *144*(2), 227–237. https://doi.org/10.1093/TOXSCI/KFU312

Savitzky, A., & Golay, M. J. E. (1964). Smoothing and Differentiation of Data by Simplified Least Squares Procedures. *Analytical Chemistry*, *36*(8), 1627–1639. https://doi.org/10.1021/AC60214A047/ASSET/AC60214A047.FP.PNG_V03

Sirenko, O., Cromwell, E. F., Crittenden, C., Wignall, J. A., Wright, F. A., & Rusyn, I. (2013). Assessment of Beating Parameters in Human Induced Pluripotent Stem Cells Enables Quantitative In Vitro Screening for Cardiotoxicity. *Toxicology and Applied Pharmacology*, *273*(3), 500. https://doi.org/10.1016/J.TAAP.2013.09.017

van Hasselt, J. G. C., Rahman, R., Hansen, J., Stern, A., Shim, J. V., Xiong, Y., Pickard, A., Jayaraman, G., Hu, B., Mahajan, M., Gallo, J. M., Goldfarb, J., Sobie, E. A., Birtwistle, M. R., Schlessinger, A., Azeloglu, E. U., & Iyengar, R. (2020). Transcriptomic profiling of human cardiac cells predicts protein kinase inhibitor-associated cardiotoxicity. *Nature Communications*, *11*(1). https://doi.org/10.1038/S41467-020-18396-7

Wu, T., Hu, E., Xu, S., Chen, M., Guo, P., Dai, Z., Feng, T., Zhou, L., Tang, W., Zhan, L., Fu, X., Liu, S., Bo, X., & Yu, G. (2021). clusterProfiler 4.0: A universal enrichment tool for interpreting omics data. *Innovation (Cambridge (Mass.))*, *2*(3). https://doi.org/10.1016/J.XINN.2021.100141

Yang, H., Stebbeds, W., Francis, J., Pointon, A., Obrezanova, O., Beattie, K. A., Clements, P., Harvey, J. S., Smith, G. F., & Bender, A. (2022). Deriving waveform parameters from calcium transients in human iPSC-derived cardiomyocytes to predict cardiac activity with machine learning. *Stem Cell Reports*, *17*(3), 556–568. https://doi.org/10.1016/J.STEMCR.2022.01.009
